# Metal nitride-based nanostructures for electrochemical and photocatalytic hydrogen production

**DOI:** 10.1080/14686996.2022.2029686

**Published:** 2022-03-14

**Authors:** Harpreet Singh Gujral, Gurwinder Singh, Arun V. Baskar, Xinwei Guan, Xun Geng, Abhay V. Kotkondawar, Sadhana Rayalu, Prashant Kumar, Ajay Karakoti, Ajayan Vinu

**Affiliations:** a Global Innovative Centre for Advanced Nanomaterials (GICAN), College of Engineering, Science and Environment (CESE), School of Engineering, The University of Newcastle, University Drive, Callaghan, 2308, Australia; b Environmental Materials Division, CSIR-National Environmental Engineering Research Institute, Nehru Marg, Nagpur, 440020, India

**Keywords:** Metal nitrides, porous metal nitrides, porosity, electrochemical hydrogen production, photocatalytic hydrogen production, 50 Energy Materials, 102 Porous / Nanoporous / Nanostructured materials < 100 Materials, 103 Composites < 100 Materials, 205 Catalyst / Photocatalyst / Photosynthesis < 200 Applications, 301 Chemical syntheses / processing < 300 Processing / Synthesis and Recycling, 501 Chemical analyses < 500 Characterization, 502 Electron spectroscopy < 500 Characterization

## Abstract

The over-dependence on fossil fuels is one of the critical issues to be addressed for combating greenhouse gas emissions. Hydrogen, one of the promising alternatives to fossil fuels, is renewable, carbon-free, and non-polluting gas. The complete utilization of hydrogen in every sector ranging from small to large scale could hugely benefit in mitigating climate change. One of the key aspects of the hydrogen sector is its production via cost-effective and safe ways. Electrolysis and photocatalysis are well-known processes for hydrogen production and their efficiency relies on electrocatalysts, which are generally noble metals. The usage of noble metals as catalysts makes these processes costly and their scarcity is also a limiting factor. Metal nitrides and their porous counterparts have drawn considerable attention from researchers due to their good promise for hydrogen production. Their properties such as active metal centres, nitrogen functionalities, and porous features such as surface area, pore-volume, and tunable pore size could play an important role in electrochemical and photocatalytic hydrogen production. This review focuses on the recent developments in metal nitrides from their synthesis methods point of view. Much attention is given to the emergence of new synthesis techniques, methods, and processes of synthesizing the metal nitride nanostructures. The applications of electrochemical and photocatalytic hydrogen production are summarized. Overall, this review will provide useful information to researchers working in the field of metal nitrides and their application for hydrogen production.

## Graphical abstract

**Figure:**
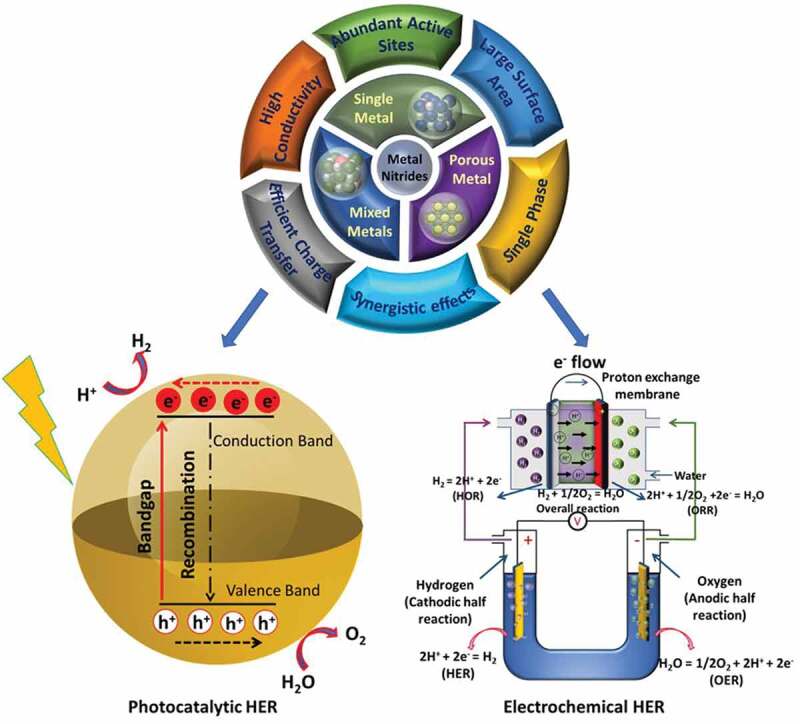

## 1. Introduction

Fossil fuels–based energy generation is accompanied by greenhouse gas emissions, which is a major concern for global warming and climate change. The over-dependence on such resources and their continuous depletion creates the urgent demand for finding alternative solutions, which can provide a continuous and competent amount of energy as compared to conventional fossil fuels. Hydrogen is deemed to be one of the cleanest alternative sources of energy, which, however, is not readily available. Hydrogen is a highly effective source of energy and can deliver a very high mass-energy density of 120 MJ kg^−1^, which is close to five times the energy of fossil fuels such as coal [1]. Steam reforming is one of the major technologies for the industrial production of hydrogen. However, the accompanied emissions pose concerns for the environment [2,3]. Water, as the simplest of the molecules, contain 11.11 wt % of hydrogen, which along with its natural abundance makes it the pivotal theme of enormous research undertaken for hydrogen production by using either the electrochemical or the photocatalytic pathways [4,5]. These two methods of producing hydrogen are more sustainable as compared to the industrial steam reforming process for hydrogen generation as these methods are more sustainable and cause no damage to the environment. Although the electrochemical pathway to produce hydrogen via electrolysis of water is a well-established method on a commercial basis, photocatalytic water splitting stands out for more sustainable production of hydrogen from solar power, which is a renewable source of energy. A catalytic material is essential to drive the splitting of water and noble metals have been the preferential choice for this purpose owing to their high catalytic activity [6,7]. However, the primary drawbacks of employing noble metals are their high cost and low natural abundance [8]. Therefore, it is highly significant to develop non-noble metal electro- and photo-catalysts that are economical, available in large quantities, and highly efficient for producing hydrogen from water.

The existing literature is flooded with numerous catalytic materials for electrochemical and photocatalytic splitting of water and including metals supported on porous materials [9], metal-organic frameworks (MOFs) [10], conjugated polymers [11], hybrids [12], composite metal oxides [13], nitrogen doped carbon and carbon nitrides [14–30], and metal nitrides (MNs) [31], etc. Among these catalytic materials, MNs have evolved extremely promising owing to their exquisite characteristic features such as high electrical conductivity, high structural stability under extreme thermal conditions, and corrosion resistance [32]. For example, MNs of molybdenum [33] and titanium [34] have widely been reported for their high efficiency for hydrogen production. MNs, in general, possess high electron density, thermal and chemical stability, structural stability and adjustable band gap which are favorable factors for hydrogen production through either electrochemical or photocatalytic pathways. The preferred method of synthesizing the MNs is from the thermal annealing of metal oxides or similar compounds in the presence of ammonia [35]. The nitridation process is generally carried out in the presence of ammonia, however, on occasions, few other sources of nitrogen such as urea have been employed to address the issue of toxicity associated with the use of ammonia. Over the years, different variants of MNs, including single MNs [36], bimetallic nitrides [37], and ternary metallic nitrides [38], have been developed for the hydrogen production sector. It is envisaged that the catalytic activity of the MNs is imparted by the metal centre and the nitrogen atom [39,40]. Porosity is another key factor that enhances the number of active sites for electro- and photocatalysis. For example, one of the fascinating aspects of the materials such as carbons, MOFs and zeolites is their porosity, which imparts properties such as high surface area, large pore volume, and tunable pore sizes, leading to improved application efficacies. Occasionally, MNs have also been explored for their porous nature, however, the reported surface area is only low to moderate [41], owing to the large atomic weight of the elements in the nitrides, and the metal and nitrogen are not inherently suited for the generation of porosity. Some reports strongly suggest that the presence of porous features in MNs result in higher catalytic activity. For instance, porous molybdenum nitride with a surface area of 197.4 m^2^ g^−1^ displayed remarkable performance due to its porosity and high conductivity [42]. The introduction of porosity is one of the crucial aspects to elevate their performance, and it could be accomplished mainly by using thermal annealing in combination with other methods such as reactive templating, porous supports, molten salt route etc.

**Figure 1.**
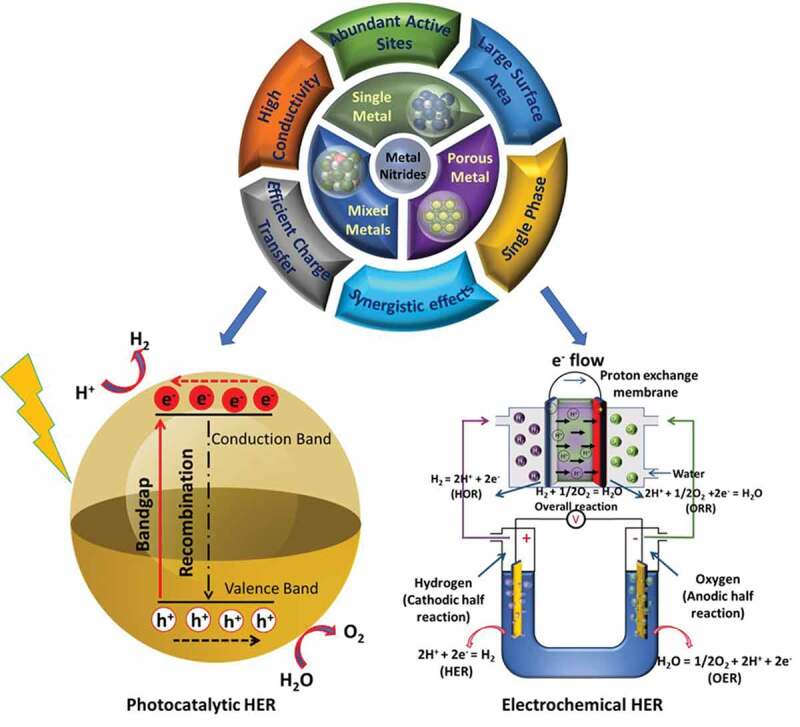
Illustration of the synthesis, structure and application of MNs for hydrogen production.

Overall, MNs are a unique class of materials that have shown great potential for electrochemical and photocatalytic pathways to generate hydrogen. The MNs could be synthesized in a single or multi-metal combination and perfected by varying experimental conditions for enhancing their application efficacy. A graphical illustration of various synthesis methods, structures and applications for hydrogen generation of different types of MNs is provided in Figure 1. Although the field is rapidly evolving, there are still challenges associated, of which the major one is to eliminate the use of ammonia for nitridation by employing alternative chemicals, which are environmental-friendly. Another focus that has been addressed on very few occasions in the past is the synthesis of MNs at relatively low temperatures by eliminating the use of the thermal annealing process [43]. This review covers the synthesis of MNs in a single and multimetallic combination in both non-porous and porous states. The fundamentals and the different methods covered for the synthesis will provide readers with a comprehensive overview of the field and offer insight into the further development of MNs via more environmentally benign methods. The application perspective of the MNs for electrochemical and photocatalytic hydrogen production is discussed in detail to demonstrate the suitability of the MNs for these applications. Recently, there has been a lot of reports on the introduction of porosity in MNs and their impact on the final electro and photocatalytic activity of the materials, especially for hydrogen production. However, there has been no reviews published on this topic so far. Hence, the current review is a timely presentation of the recent literature covering the synthesis of MNs in both non-porous and porous forms and their application in the electrochemical and photocatalytic production of hydrogen.

## 2. Synthesis of MNs

**Figure 2.**
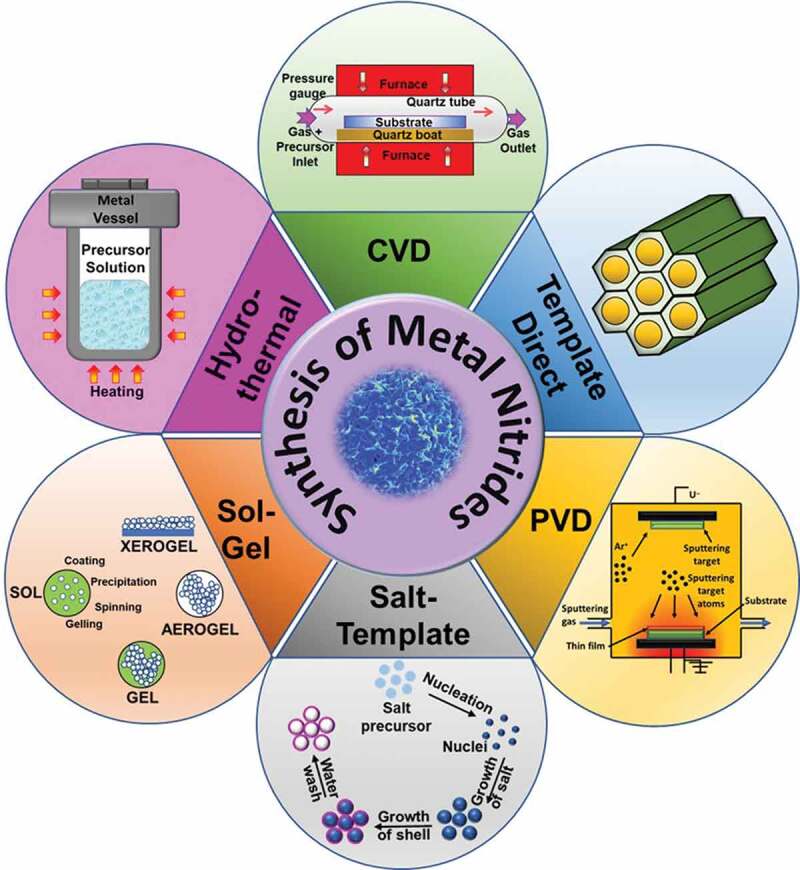
Different methods for the synthesis of MNs.

Synthesis approaches play a critical role in controlling the structure, morphology, crystallinity, and electrochemical properties of the MNs [44]. In this section, we will discuss the different methods available for the synthesis of mono and bimetallic nitrides and the functionalized MNs, including heteroatom doping and hybridization. Various synthesis procedures have been developed for the synthesis of monometallic and bimetallic nitrides [45], and can be broadly classified into physical and chemical methods of synthesis. The physical methods present general and simple strategies to synthesize highly pure and doped MN. The most common examples of physical methods of synthesis include physical vapor deposition (PVD) [46,47] and plasma/laser-based methods [48]. However, the development of multiple hybrids with different morphologies and porosities through physical methods is challenging. In contrast, the chemical methods of synthesizing MN involve both top-down and bottom-up approaches and are widely popular. For example, a range of different structures, including 0D, 1D, 2D, and 3D geometry in MN, can be introduced with the simple adjustment of the synthesis methods [49]. Similarly, heteroatom doping, defect engineering, alloying, and hybridization are employed to attain a desired structure–property relationship that is also enabled through various synthesis routes, as shown in Figure 2. These methods allow greater flexibility of synthesis as they give a wide option of synthesis of various structures and morphologies and the possibility to integrate hybridization and doping by subtle modification of the synthesis parameters. The chemical methods, however, suffer from challenges such as the inhomogeneity in size, shape and properties, use of toxic precursors, high temperature (ranging between 1000°C and up to 2000°C) and non-scalable methods. Several well-established chemical approaches are employed for the preparation of MN including controlled atmosphere annealing, template-directed methods, chemical vapor deposition, and salt-templating. Table 1 summarizes some of the MNs and their synthesis methods. In the below section, we will describe the processes involved in these approaches.

**Table 1.** Summary of the various aspects of the synthesis of various MNs

Material	Synthesis			Structural features	Ref.
	Precursors	Method	T (°C)		
W_2_N_3_	Na_2_WO_4_.2H20, K2SO4, HCl	Hydrothermal	180	3D hexagonal, flower structure	[53]
		Thermal annealing under NH_3_	700		
Ni_3_N/Ni/NF	Ni(NO_3_)_2._6H_2_O, HMT, N_2_H_4._H_2_O, NaOH, HCl, NF	Hydrothermal	100	Hexagonal, nanosheet	[72]
		Thermal annealing under NH_3_	380		
Ni_3_N/C cloth composite	Ni(NO_3_)_2._6H_2_O, HNO_3_	Hydrothermal	120	3D, nanosheet	[73]
		Thermal annealing under NH_3_	400		
PdN	Na_2_PdCl_4_, C_6_H_8_O_6_,AA, PVP (Polyvinylpyrrolidone), KBr	Hydrothermal-Nitridation	180	Nanocubes	[74]
TiN	Ti(C_4_H_9_O)_4_, C_3_H_8_O, C_3_H_8_O_3_	Solvothermal	180	2D, leaf-like nanorods	[75]
		Thermal annealing in air	450		
		CVD under NH_3_	800		
TiN	Sol 1 – TIPT, BzAc, MeOH	Sol-gel thermal nitridation	110	Cubic	[76]
	Sol 2 – TIPT, H_2_O, HCl, BuOH				
WN	WO_3_, NaCl, SiO_2_/Si	CVD under NH_3_	800	2D nanosheet	[107]
W_2_N_3_	(NH_4_)_6_H_2_W_12_O_40_·H_2_O, KCl, ethanol	salt-templating method	750	2D hexagonal, flakes	[108]
TiN	TiCl_4_	CVD under Ar and NH_3_	900–1000	2D, single crystal	[305]
ZrN	ZrCl_4_, urea, ethanol	Urea-glass route	800	Spherical	[306]
TiN	Ti(C_4_H_9_O)_4_, acetic acid	Hard template, hydrothermal	140	Hollow nanosphere	[69]
		Thermal annealing under NH_3_	800		

**Note: T**- Temperature, W_2_N_3 –_ Tungsten Nitrides, Na_2_WO_4_.2H_2_O – Sodium Tungstate Dihydrate, K_2_SO_4_ – Potassium Sulfate, HCl – Hydrochloric Acid, Ni_3_N – Nickel Nitrides, Ni(NO_3_)_2._6H_2_O – Nickel(II) Nitrate Hexahydrate, HMT- Hexamethyleneteramine, N_2_H_4._H_2_O – Hydrazine monohydrate, NaOH – Sodium Hydroxide, NF – Nickel form, HNO_3_ – Nitric acid, PdN – palladium nitrides, Na_2_PdCl_4 –_ Sodium tetrachloropalladate, C_6_H_8_O_6 –_ Ascorbic acid, KBr – Potassium bromide, TiN – Titanium Nitrides, Ti(C_4_H_9_O)_4_ – Titanium butoxide, C_3_H_8_O – Isopropyl, C_3_H_8_O_3_ – Glycerol, TIPT – Tetraisopropylorthotitanate, BzAc – Benzoylacetate, MeOH – Methyl alcohol, BuOH – Butyl alcohol, WN – Tungsten Nitrides, WO_3_ – Tungsten trioxide, NaCl – Sodium Chloride, SiO_2_/Si – Silicon dioxide/ Silicon, (NH_4_)_6_H_2_W_12_O_40_·H_2_O – Ammonium Metatungstate Hydrate, KCl – Potassium Chloride, TiCl_4_ – Titanium Tetrachloride, ZrN- Zirconium Nitrides, ZrCl_4_- Zirconium Tetrachloride, Ti(C_4_H_9_O)_4_-Titanium butoxide.

### 2.1 Controlled atmosphere annealing

Controlled atmosphere annealing is one of the most common methods for the fabrication of MN nanoparticles with different morphologies such as nanorods [50], nanowires [51], nanotubes [52], flowers shape [53], coral shape [54], and hollow nanospheres and a high crystallinity [55]. This method allows the controlled transport of the key elements as well as protects the samples from excessive heating and exposure to air at a high temperature, and has been applied for the synthesis of MN with 0D, 1D, and 2D nanostructures from their corresponding metal oxides [49]. The morphologies of these nanostructures can be finely tuned with the simple adjustment of the process conditions such as the reaction time, the reaction temperature and the reaction pressure [56]. In a typical synthesis of MNs, thermal annealing of metal oxide or other forms of the metal precursor is carried out in the presence of nitrogen-rich precursors (gases) such as ammonia [57], urea [58], and dicyanamide [59,60]. These metal oxides or other precursors are usually synthesized using hydrothermal [61], sol-gel [62], chemical precipitation [43], electrospinning [63,64], electrodeposition [65], or solvothermal methods [66]. The morphology and high crystallinity of metal oxide precursors fabricated during the hydrothermal synthesis [67] are directly transferred to the MN upon annealing under nitrogen-rich conditions. However, the change in the volume of the material during its conversion from metal oxide or hydroxide to MN often results in agglomeration or pulverization of the fabricated MN that needs to be strictly controlled in order to achieve the desired structure of MN.

As suggested above, the conversion of metal oxides to MNs by heating in an ammonia atmosphere at moderately high temperatures is a popular method to produce MNs with different morphologies [68,69]. Recently, Tan et al. [53] developed a strategy to form 3D tungsten nitrides (W_2_N_3_) from tungsten oxide at high temperatures using ammonia as a nitrogen source. Tan and the team first prepared a crystalline hexagonal tungsten trioxide (WO_3_) precursor with 3D flower-like morphology using the hydrothermal process at 180°C for 12 h. The 3D structure was obtained by the addition of potassium ions in the hydrothermal synthesis mixture as these ions enable the growth of flower-like morphology. The WO_3_ nanomaterial was further converted to flower-like W_2_N_3_ by annealing in an ammonia atmosphere for 2 h at 700°C. The nitrogen-rich, micron-sized motifs exhibited high structural stability while retaining the 2D sheet-like morphology within the flower structures. As the 3D flower structure, generated from the WO_3_ nanostructures, is stabilized by 2D nanosheets of W_2_N_3_, this unique material exhibits the properties of both 3D and 2D nanostructures. Similarly, Ni-based MNs that are widely studied for energy storage and conversion, have also been synthesized predominantly using the thermal annealing approach [70,71]. Lin et al. [72] fabricated highly active and well-dispersed nickel nitride (Ni_3_N) nanoparticles scattered on the porous nickel (Ni) foam. At first, hexagonal nickel hydroxide nanosheets were grown over the Ni foam via the hydrothermal method followed by nitridation in ammonia/argon atmosphere at 380°C for 5 h to complete the nitridation procedure. The nanosheet-like architecture was retained after the nitridation process. However, distinct microstructural and phase changes were observed wherein the Ni(OH)_2_ was successfully converted to hexagonal Ni_3_N.

**Figure 3.**
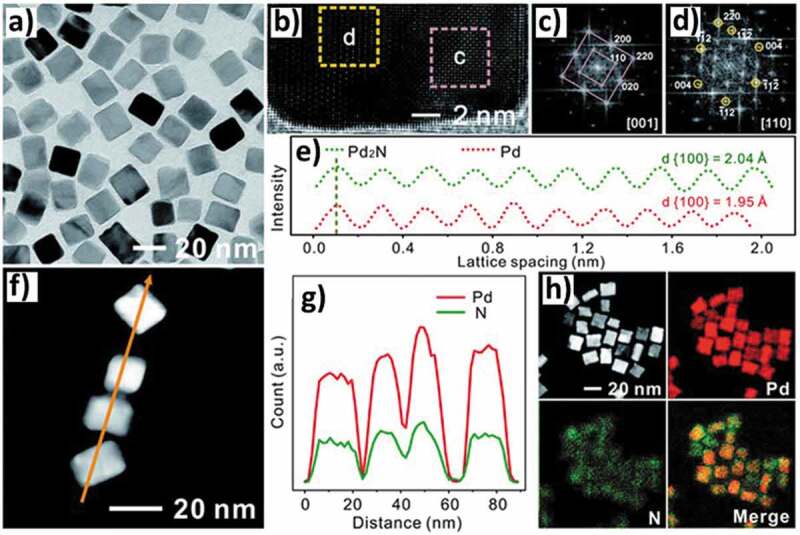
Morphological characterization of tetragonal Pd_2_N nanocrystals. (a) Low-magnification TEM image of Pd_2_N nanocrystals. (b) High-resolution TEM (HR-TEM) image of a typical Pd_2_N nanocrystal. (c and d) Fast Fourier transform (FFT) patterns taken from the pink and Orange dotted squares in (b) marked as c and d. (e) The integrated pixel intensities of Pd nanocubes and Pd_2_N nanocrystals along (100) spacing directions and the integrated pixel of Pd_2_N nanocrystals was taken from the pink dotted square area c in (b). (f) High-angle annular dark-field scanning TEM (HAADF-STEM) image of Pd_2_N nanocrystals with the Orange line showing the scanning path and (g) the corresponding line-scanning profile. (h) HAADF-STEM image and corresponding elemental mappings of Pd_2_N nanocrystals (Reproduced with permission from [74]).

MNs based on noble metals were similarly synthesized using a combination of hydrothermal and thermal annealing processes. For example, Balogun et al. [73] demonstrated the synthesis of flexible 2D Nickel nitrides on a 3D carbon cloth forming 3D Ni_3_N/ carbon composite cloth using the hydrothermal and post-annealing method. The morphological, structural, and metallic features of the 2D Ni_3_N nanosheets offer excellent electronic conductivity. On the other hand, Guo et al. [74] reported the synthesis of non-stoichiometric palladium nitrides (PdN_x_) nanocrystals using Pd nanocubes through a simple hydrothermal decomposition of the urea precursors. The stoichiometry could be controlled by varying the weight ratio of Pd nanocubes to urea while thermal annealing in nitrogen atmosphere successfully inserted the N within the Pd lattice structure while maintaining the cube-like morphology (Figure 3). The interstitial incorporation of nitrogen atoms increases the interaction between the Pd and the nitrogen atoms which is critical for improving the electrocatalytic performance as it significantly decreases the d-band centre of Pd. It is also worthwhile to note that the increase in nitrogen content increases the catalytic performance of PdN_x_ nanocubes. In another work, Hou et al. [75] reported the synthesis of chrysanthemum-like TiN from titanium dioxide (TiO_2_) synthesized using the solvothermal alcoholysis method through a reduction and nitridation process at 800°C in NH_3_ atmosphere for 2 h. The 2D chrysanthemum-like morphology comprising of 2D leaf-like nanorods of TiO_2_ could be preserved during the transformation to TiN by controlling the heating rate. However, the volume changes results in the creation of mesoporous architecture formed by the stacking of cubic TiN nanoparticles in the form of a leaf-like structure. This unique structure is very efficient for the ion diffusion as well as offers good structural stability during charging and discharging of the supercapacitor electrodes fabricated from this material.

The combination of thermal annealing with other methods of synthesis is not only restricted to solvothermal and alcohothermal-based synthesis but metal oxides or precursors synthesized by any other method can also be converted to MN by thermal treatment under a nitrogen containing precursors. For example, Ti-based nitrides were synthesized by Valour et al. [76] by combining the thermal annealing in ammonia with the sol-gel photo patternable films of TiO_2_. The resulting highly crystalline TiN with a thickness of 60 nm was prepared within a short time and was more scalable and economical as compared to other methods of synthesis. Mahdi Safa and his team compared different nanosized TiN nanoparticles prepared using the ball milling, sol-gel, and co-precipitation methods [77]. In both co-precipitation and sol-gel methods, the resulting material synthesized using these processes were directly annealed in the ammonia atmosphere for 5 h at 1000°C to obtain TiN nanoparticles. The XRD pattern of TiN indicated that annealing at 1000°C results in the complete formation of TiN while the ball milling samples had traces of TiO_2_ even after milling at 500 rpm. Annealing at lower temperatures (700–800°C) also resulted in the incomplete transformation of TiO_2_ to TiN, suggesting the importance of temperature in the thermodynamically unfavorable conversion of TiO_2_ to TiN. In a similar approach, metallic Ni_3_N nanosheets with an average sheet thickness of less than 3 nm were prepared from NiO nanosheets by heating at 380°C under a flow of ammonia [78]. The prepared nanosheets display graphene-like behavior with extremely high electrical conductivity and disordered structure, which are useful for improving the electrochemical performance in oxygen evolution reaction.

### 2.2 Template-directed methods

The template-directed method of synthesis is one of the most useful methods to obtain controlled morphology along with the uniform shape and size of MN nanoparticles. The template methods are broadly classified into two categories – hard and soft templating [19,23–26,79–93]. Hard templating is usually shape-defining physical scaffolds that are synthesized prior to the final synthesis of MN and serve as the templates for the deposition of the precursors in a conformal manner over the template or inside the porous or other intricate structures [94]. The unique morphology and stable structure of the templates play an important role in applications where such replication of particle structure is necessary [95]. However, the removal of the hard template is fairly challenging and has been substituted by reactive templating methods where the template is consumed during the reaction or synthesis of materials [96]. On the contrary, soft templates do not have a fixed rigid structure and the templates are developed in situ, during the reaction via intermolecular or intramolecular interactions. The soft templating is primarily based on the self-assembly of a surfactant or polymeric micelles which are assembled into different organic-inorganic phases depending upon the interaction between ligands, surfactants, polymers, organogelators, and target precursors of the final catalytic and adsorbent materials as well as temperature and concentration of materials [97–100] The narrow stability of pH and solvents limit the application of self-assembly in its synthesis. Template-based methods are primarily used in the synthesis of porous TiN discussed later in this review and a few examples of hollow TiN are discussed in this section.

**Figure 4.**
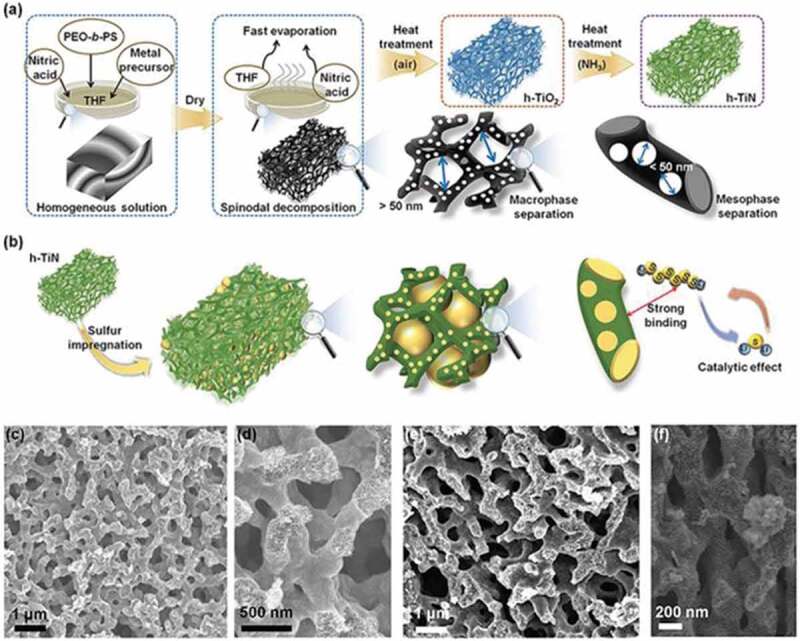
A,b) Schematic illustrations for the synthetic route of co-continuous h-TiN (a) and its application as a host material for sulfur (b). The nitric acid and controlled evaporation (NICE) process induce macrophase separation via spinodal decomposition (SD). Meanwhile, the evaporation-induced self-assembly (EISA) method is widely used to synthesize the mesoporous structure via phase separation between a hydrophobic polymer block and a hydrophilic polymer/inorganic precursor block. c–f) SEM images of h-TiO_2_ (c,d) and h-TiN (e,f). The hierarchical multiscale porous structure is still retained without any collapse after the conversion to h-TiN. The good retention of the porous structure is attributed to the thick pore wall of the h-TiO_2_ derived from the block copolymer self-assembly (Reproduced with permission from [104]).

Li et al. [101] reported the synthesis of TiN hollow nanospheres of ~160 nm diameter using porous silica spheres as a template. In this process, TiO_2_ nanospheres were prepared from SiO_2_ nanospheres that were further converted to TiN by heating at 800°C for 2 h in an ammonia atmosphere. Further, the TiN hollow spheres were combined with sulfur via the melt-diffusion method to form hollow TiN-S composites that showed exceptional performance in the Li-S batteries. Mesoporous carbon can also be used as a reactive template for the synthesis of hollow spheres of TiN. The residual carbon is activated during the calcination process and doped with nitrogen, presenting tremendous carbon, and nitrogen active sites for electrochemical reactions. TiO_2_ was first synthesized over the carbon structures via sol-gel processing using tetrabutyl titanate followed by a hydrothermal process at 180°C for 6 h. The as-synthesized c@TiO_2_ spheres were calcined in air to remove carbon (partially or fully) and converted to TiN nanosheets by calcination at 900°C in ammonia atmosphere for 2 h. The prepared TiN hollow sphere assembled by 2D nanosheets of TiN exhibited high surface areas of 316.7–358.3 m^2^/g, depending upon the calcination treatment, and a uniform ~30 nm shell thickness depicting a combined advantage of the unique structure and more active sites [102]. Similarly, nanotemplating was used to prepare nanocrystalline 3D porous boron nitride foams at 1150°C using porous sacrificial polyHIPEs as the soft template [103]. In contrast, in a soft-templating approach (Figure 4), Lim et al. demonstrated that by controlling the microsphere and mesophase separation using spinodal decomposition and self-assembly methods, respectively, porous TiN nanoparticles can be developed via a one-pot method instead of the hard templating approach [104]. The prepared TiN exhibits excellent electrical conductivity and mechanical strength and hierarchical structure with the combination of both macropores and mesopores in a single system. These unique surface features and electronic properties of TiN can help to accommodate large amount of sulfur and also facilitate the easy penetration of the electrolyte and the transportation of alkali metal ions for the energy storage devices.

### 2.3 Chemical vapor deposition

**Figure 5.**
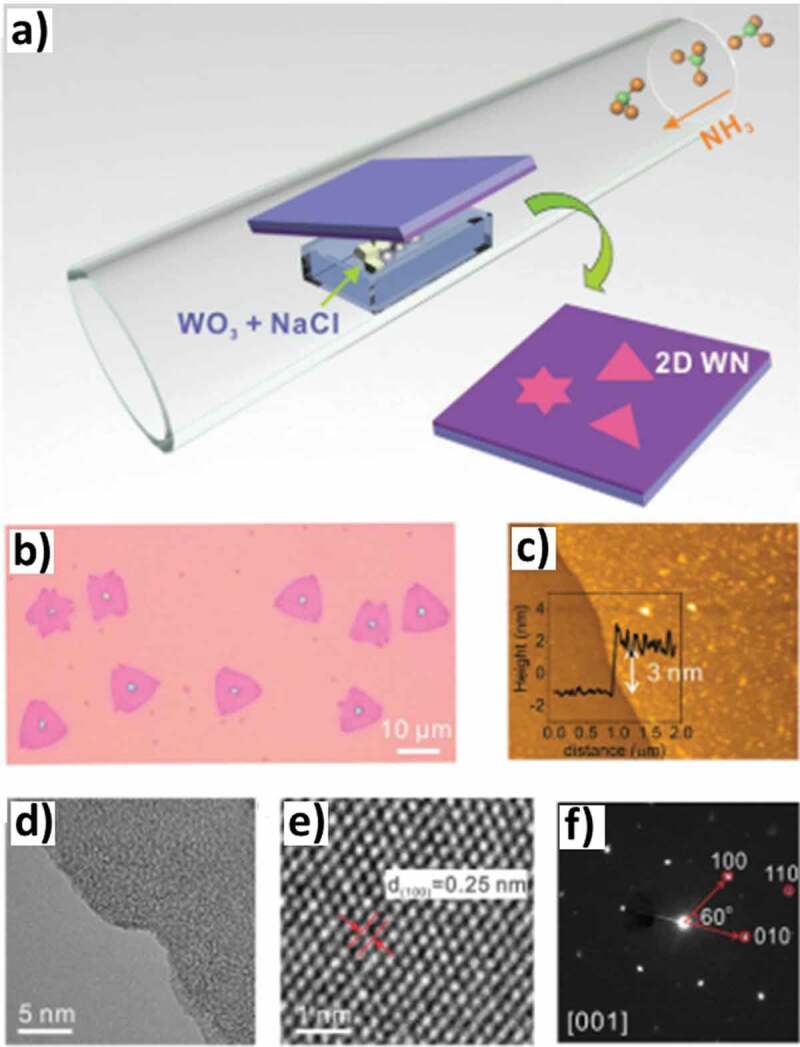
A) Illustration of the CVD synthesis of ultrathin WN. b) Optical image of ultrathin WN crystals deposited on a SiO_2_/Si substrate. c) AFM measurement indicates the typical thickness of WN is 3 nm (inset: AFM height profile). d) A TEM image shows the edge of a transferred ultrathin WN layer. e) A high-magnified TEM image shows the (100) lattice of WN. f) SAED pattern of WN (Reproduced with permission from [107]).

Chemical vapor deposition (CVD) is a popular method for the synthesis of a range of materials of various shapes, sizes, and morphologies including single or bimetallic compositions of MN. In the CVD approach, a chemical reaction is induced in the vapor phase at various temperatures and low-pressure accompanied by the deposition of the final material on the substrate. The process is versatile and can be applied to form multiple types of compounds including but not limited to the involvement of the substrate. For example, Ji et al. [105] prepared an integrated strategy to synthesize large monolayer hexagonal boron nitride (h-BN) on the surface of polycrystalline copper foil by the low-pressure CVD technique. The author also demonstrated the fabrication of monolayer, bi-, and tri-layer hexagonal BN structures using this technique. The boron and nitrogen were brought in the vapor phase by heating ammonia borane at ~70°C and fed in the CVD reactor to initiate the growth of hBN at 1050°C under H_2_ flow over an electropolished Cu foil. The Cu substrate orientation and the conditions for electropolishing influenced the morphology of the monolayers. It was also observed that the bilayers of different morphologies including triangle, trapezoid, and hexagon, could be achieved over monolayers. Similarly, Chang et al. [106] synthesized an h-BN using ammonia borane precursor on the surface of Cu foil with a thin passivating oxide layer as substrate. Similar to the report of Ji et al. [105], the epitaxial relationship between the Cu foil and the h-BN leads to the formation of large-sized crystalline domains of approximately 1–20 μm within the ∼100 μm film. Other materials such as tungsten nitride (WN) have also been grown using CVD technique. For example, Wang et al. [107] synthesized ultrathin single-crystal WN using the salt-assisted chemical vaporization deposition technique on a silica substrate as shown in Figure 5. WN was synthesized by evaporation of WO_3_ premixed with NaCl from an alumina boat within the tube furnace followed by the introduction of ammonia (NH_3_) for the formation of WN. The resulting h-WN films were triangular in orientation and around 3 nm in thickness. It was found that films do not grow in the absence of salt, suggesting the role of salt in the formation of intermediate compounds that are more volatile and facilitate the deposition of WN films. The structure could also be tuned by varying the concentration of ammonia introduced in the systems and an increase in the ammonia concentration leads to the transition of 2D ultrathin WN to 3D nanostructures, suggesting the versatility of this approach. Similarly, syntheses of many other transition MNs with different structures including 0D, 1D, and 2D using the CVD technique and the strategies of manipulating the electronic properties through doping, defect engineering and hybridization have been previously reviewed by Wang et al. [49].

### 2.4 Salt-templating strategy

**Figure 6.**
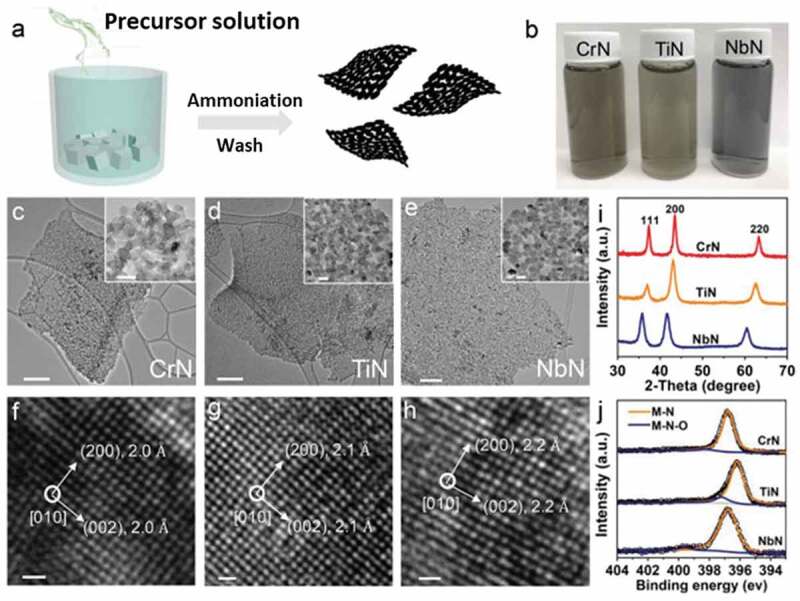
Synthesis and characterizations of 2D arrays of TMN nanocrystals. a) Schematic of synthesis. The precursor solution in ethanol was poured into 100 g of a salt template. After stirring and drying in an oven at 70°C, a thin layer of the precursor was formed on the surface of salts (labeled as precursor@salt). Then the precursor@salt powder was treated in a furnace at 700–750°C under a constant flow of ammonia. Finally, after dissolving the salt template in deionized water (which can be collected and recycled), 2D arrays of TMN nanocrystals were obtained. b) Digital optical images of colloidal solutions of TMN nanocrystals dispersed in deionized water. c–e) TEM images of the 2D arrays of TMN nanocrystals. Scale bars are 200 nm for (c) and (e), and 500 nm for (d). The insets show that 2D flakes are made of interconnected TMN nanocrystals. Scale bars are 10 nm. f–h) HRTEM images of TMN nanocrystals shown in (c–e). Scale bars are 5 Å. i) XRD patterns of 2D arrays of TMN nanocrystals. j) N 1s X-ray photoelectron spectra (XPS spectra) of 2D arrays of TMN nanocrystals (Reproduced with permission from [109]).

Salt-templating is emerging as one of the popular strategies for the synthesis of MN, especially 2D transition MN. In this technique, which is an extension of the topochemical synthesis method, metal precursors are ammoniated on the salt surface while keeping the epitaxial relationship during the synthesis. 2D hexagonal N-rich W_2_N_3_ nanosheets have been reported by the ammonization of (NH_4_)_6_H_2_W_12_O_40_·*x*H_2_O pre-coated on the surface of KCl at 750°C [108]. The formation of W_2_N_3_ nanosheets was facilitated by the epitaxial relationship with KCl and the interfacial energy between the KCl and growing W_2_N_3_ can be reduced by introducing patterns. It was also found that the (002) crystal facet is thermodynamically most favorable for the growth of h-W_2_N_3_ over the KCl surface. Similarly, Xiao et al. demonstrated the fabrication of different types of 2D transition MN following the salt templating strategy (Figure 6) [109]. In this unique process, the growing transition MN on the surface of the salt template was etched and recrystallized during the ammonization process, resulting in the formation of continuous 2D arrays. The process was reproducible and was appropriate for the synthesis of CrN, TiN and NbN 2D nanoflakes that were composed of interconnected nanocrystals. In a unique method, a stable 2D Mo_5_N_6_ structure was reported by Jin et al. using the salt templating strategy that was assisted by nickel [110]. It was shown that incorporation of nickel during the salt templating method along with molybdenum precursors helps in the formation of N rich molybdenum nitrides while in the absence of Ni a nitrogen deficient phase of pure 2d MoN is achieved. The nickel is reduced to metallic nickel under an ammonia atmosphere and is dissolved by acid treatment to yield nickel doped Mo_5_N_6_ flakes. It must be noted that the addition of Ni also helps in reducing the nitridation temperature of NiMoN from 850°C to 550°C for the synthesis of pure MoN.

It is important to mention that control of the topology, morphology, crystal structure, and the surface and electronic properties of the MN is critical as these factors have a direct relation with their electrochemical performance. Therefore, it is crucial to choose the right method to tune and control the structure and properties of these MN nanostructures to enhance electrochemical performance. Although many synthesis strategies are available, templating strategies are the unique approach to finely control both the structure and morphology and the electrochemical properties of the nanostructures. These methods are also versatile as they allow the easy manipulation of the architecture, composition, electronic structure, doping, and other modification. However, in the future, the integration of these synthesis strategies may be required to advance the design and development of MNs with much better surface and electrochemical properties.

## 3. Synthesis of mixed MNs

Earlier studies reveal that single MNs might suffer from severe issues related to electrochemical instability in alkaline or acid conditions [111–113], which can cause a decrease in performance efficiency. To address these issues, strategies like doping and substituting with multiple elements are potential solutions that have prompted the development of exciting materials in the category of mixed MNs, including bimetallic or ternary nitride frameworks [38,114,115]. The partial substitution of metal elements into the material matrix, such as carbon nitrides and metal oxides, considerably modifies their crystalline structure and improves electric tunability, thereby making them more promising from the application perspective. In this subsection, a series of mixed MNs including Ni/MoN_x_ [31,116–118], Ni/CoN_x_ [119–122], Ni/FeN_x_ [123,124], Mo/CoN_x_ [125], Cu/MoN_x_ [126], V/CoN_x_ [127], and others [128–130] will be discussed primarily from their synthesis point of view. Chemical and physical routes are two main procedures adopted for the fabrication of mixed MNs [131]. The chemical route mainly focuses on heat treatment by reacting metal precursors with nitrogen sources at increased temperatures, with advantages of large-scale production and easy composition control. On the other hand, the physical route covers multiple advanced techniques, including chemical vapor deposition (CVD), magnetron sputtering, molecular beam epitaxy (MBE), and pulsed laser deposition (PLD), guaranteeing excellent crystalline quality and device performance [117,131–134]. Some representative mixed MNs and their synthesis strategies have been summarized in Table 2.

**Table 2.** Summary of material synthesis methods for mixed MNs

Composition	Synthesis method	Nitridation source/ Temperature (^o^C)	Ref.
2D Mo_2_N/V_2_N	Thermo condensation	NH_3_/600	[136]
NiMoN_x_/C	Thermo condensation	NH_3_/700	[116]
NiMo_4_N_5_	Thermo condensation	NH_3_/500	[117]
Ni_3_FeN	Hydrothermal-calcination	NH_3_/500	[137]
Ni_3_N-NiMoN	Hydrothermal-calcination	NH_3_/500	[118]
Fe_2_Ni_2_N	Hydrothermal-calcination	NH_3_/380	[138]
NSP-Ni_3_FeN	Hydrothermal-calcination	Urea/150	[139]
NiCoN	Hydrothermal-calcination	NH_3_/500	[119]
VN-Co-P	Hydrothermal-calcination	NH_3_/600	[140]
FeNi_3_N/NF	Ammonolysis	NH_3_/350-450	[124]
Cu_x_Ni_4−x_N	Ammonolysis	NH_3_/380	[128]
FeN_x_/Mo_2_N/C	Ammonolysis	Melamine/600	[149]
Fe_3_Mo_3_N/Co_3_Mo_3_N/Ni_2_Mo_3_N	Pyrolysis	Urea-N_2_/900	[150]
Co/N-VCoN	Pyrolysis	Urea/900-1000	[127]
MoVN	Magnetron sputtering	N_2_/RT	[161]
Multi-channel TiN micro/nanotubes	Electrospinning	NH_3_/400-1000 for the precursor of nanofibers or micro/nanotubes	[159]
NiMoN	Electrodeposition	RT at a cathodic current density of 30 Ma/cm^2^ and 2 Hz frequency for 7200 cycles	[160]
Ti_x_M_1-x_N and Ta_x_M_1-x_N	Pulsed laser deposition	base pressure 10^−8^ mbar, 2/2 sccm for the Ar/N_2_ flow rate	[169]
AlGaN	MOCVD	NH_3_/850-1100	[307–309]
CdNNi_3_/InNNi_3_	Ammonolysis	NH_3_/400-600	[147]
NiMoN_x_/C	Ammonolysis	NH_3_/700	[116]
(Ti/Zr)N_x_	Magnetron sputtering	0.8 Pa Ar/N_2_/100 W/6.3–8.7 nm/min growth rate	[164]
AlGaN/ TiVN	Templating	Mixed C_3_N_4_/metal precursors/800	[155]

**Note**: NSP (Nanoparticle-Stacked Porous), MOCVD (Metal Organic Chemical Vapor Deposition), Mo_2_N (molybdenum nitride), V_2_N (vanadium nitride), Ni_2_Mo_3_N, NiMo_4_N_5_, NiMoN_x,_ Ni_2_Mo_3_N and NiMoN (nickel molybdenum nitride), C (carbon), Fe_2_Ni_2_N, Ni_3_FeN and FeNi_3_N (nickel iron nitride), NiCoN (nickel cobalt nitride), Co/N (cobalt nitride), FeN_x_ (iron nitride), MoVN (molybdenum vanadium nitride), VCoN (cobalt vanadium nitride), Fe_3_Mo_3_N (iron molybdenum nitride), Co_3_Mo_3_N (cobalt molybdenum nitride), TiN (titanium nitride), Ti_x_M_1-x_N (doped titanium nitride), Ta_x_M_1-x_N (doped tantalum nitride), AlGaN (aluminium gallium nitride), CdNNi_3_ (cadmium nickel nitride), InNNi_3_ (Indium nickel nitride), (Ti/Zr)N_x_ (titanium zirconium nitride), TiVN (titanium vanadium nitride), C_3_N_4_ (graphene-like carbon nitride), NH_3_ (ammonia gas), RT (room temperature), N_2_ (nitrogen), Cu_x_Ni_4−x_N (copper-doped nickel nitride), VN-Co-P (cobalt and phosphorus dual-doped vanadium nitride), Ar (argon gas), 2D (two dimensional).

Heat treatment (or thermo-condensation) of mixed metal precursors under ammonia atmosphere is the most scalable method, and two steps calcination process via first H_2_ reduction and subsequent NH_3_ nitridation has evolved as one of the standard routines [70,135,136]. Among MNs, nickel nitride-based materials possibly received the most attention, and these have been widely employed for electrochemical and photocatalytic applications. These materials surpass conventional noble metal electrocatalysts in HER applications thanks to their intrinsic physical properties, enabling them to bind extremely tight to both hydrogen atoms and water molecules and present low electrical resistance. In 2012, Chen et al. first reported the successful synthesis of carbon-supported NiMo nitride nanosheets (NiMoN_x_/C) by reducing carbon-contained ammonium molybdate (NH_4_)_6_Mo_7_O_24_·4 H_2_O and nickel nitrate (Ni(NO_3_)_2_·4 H_2_O) under the H_2_ atmosphere at 400°C, and subsequent nitridation with NH_3_ at 700°C, where the obtained NiMoN_x_/C nanosheets have a Ni/Mo ratio of 1/4.7 [116]. Similarly, Li<apos;>s team developed a stable biphasic Ni-Mo-N HER catalyst comprised of homogeneously distributed metallic Ni and NiMo_4_N_5_ nanocrystals [117]. The ammonium nickel molybdate precursor (NH_4_)_6_Mo_7_O_24_·4 H_2_O and Ni(NO_3_)_2_·4 H_2_O was heated with H_2_ followed by nitridation with NH_3_ at 500°C, and the fabricated device exhibited high HER performance and stability due to the unique biphasic structure combining metallic Ni-nanocrystals with an acid-stable nitride phase.

**Figure 7.**
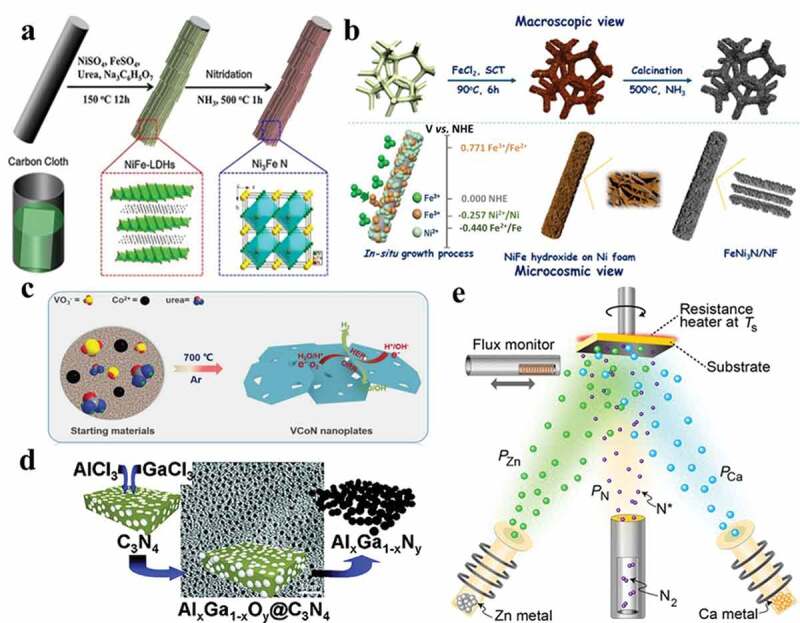
(a) Schematic of hydrothermal-calcination for Ni_3_FeN-NPs [123]. Copyright Electrochimica Acta 2017. (b) The surface-redox-etching Ni foam process of FeNi_3_N/NF (Reproduced with permission from [124]). (c) The schematic synthesis procedure for VCoN nitridated by urea (Reproduced with permission from [127]) (d) The schematic description of Al/Ga and Ti/V mixed MN nanoparticles synthesized by reactive hard templating (Reproduced with permission from [155]). (e) The RF-MBE synthesis system of CaZn_2_N_2_ films on GaN template layers (Reproduced with permission from [168]).

Other than conventional metal oxides, layered double hydroxides (LDHs) are gradually becoming another excellent precursor to prepare mixed MNs via a general approach of two-step hydrothermal-calcination [137]. In a typical procedure, mixed metal LDHs can be prepared by a hydrothermal method, and the obtained LDHs are then nitridated by NH_3_. The resultant mixed MNs can inherit the LDHs’ lamellar morphology and possess a relatively high conductivity. For instance, Chen et al. designed 3D iron-nickel nitride (Ni_3_FeN) nanoparticles with a straightforward synthesis process illustrated in Figure 7a [123]. NiFe LDHs (NiFe-LDHs) nanosheets were first grown on carbon cloth via a seed-assisted hydrothermal process and then nitrided with NH_3_ at 500°C for 1 h. Likewise, Wu and his coworkers recently adopted Ni_3_N-NiMoN heterostructures on carbon cloth for overall water splitting, exhibiting excellent catalytic activity for OER and HER [118]. In this work, the single-source Ni-Mo-O precursor of Ni_3_N-NiMoN electrocatalyst was first formed on the carbon cloth by a hydrothermal method, then calcinated under N_2_ and followed NH_3_ atmosphere at 500°C for nitridation. Such fabrication techniques have been extensively explored by different groups for other mixed MNs, such as Fe_2_Ni_2_N [138], NSP-Ni_3_FeN nanosheets [139], NiCoN nanowires [119], VN-Co-P [140], and so forth.

Bimetallic or trimetallic MNs have also been fabricated from the corresponding metal hydroxides through nitridation process [141–143]. For example, Ni_3_N/Co_2_N with flower like morphology was prepared by introducing nitrogen into the nickel-cobalt-layered double hydroxide. This simple approach offers unique hierarchical architecture that supports for the easy access to the active sites and further promotes the transfer and the dissipation of the electrochemical species [141]. In another report, molecularly thin NiFeMnN nanosheets were synthesized by stabilizing the thin molecular layers within titanium carbide nanosheets [144]. At first, the trimetallic hydroxide with a high positive charge was electrostatically hetero assembled on the hydroxyl or fluoride terminated titanium carbide nanosheet followed by nitridation under ammonia atmosphere. Using this technique, thin bilayered mixed metal hydroxide of thickness 1.1 nm was successfully converted to mixed MN of similar wall thickness, suggesting the successful conversion during the nitridation and the strong role of the titanium carbide in preventing the agglomeration during the conversion from hydroxide to nitride.

It is worth mentioning that a relatively low calcination temperature (≤ 500°C) is required when using LDHs as precursors, thanks to the presence of hydroxyl groups. For example, in 2016, Zhang et al. demonstrated an *in situ* growth of hierarchical Fe-Ni nitride observed on surface-redox-etching Ni foam [124]. Figure 7b illustrates the synthesis process for FeNi_3_N/NF accomplished using thermal ammonolysis (500°C) of NiFe hydroxide (NiFe(OH)_x_) nanosheets grown in situ on Ni foam by simple chemical precipitation reaction. The Ni foam not only functions as an electrode substrate but also as the Ni precursor from redox etching of Fe^3+^ during the precipitation process. Therefore, this approach excluded the requirement of other nickel precursors or oxidizing agents but achieved well-dispersed iron-nickel nitride nanostructures grown directly on the nickel foam surface. In another work, bimetallic copper-nickel nitride was synthesized on Ni foam based on Cu_2_O/Ni(OH)_2_ by a two-stage method [128]. The Cu_x_Ni_4−x_N electrode went through a galvanic replacement-mediated chemical precipitation synthesis first, followed by the thermal ammonolysis under an NH_3_ atmosphere only at 380°C. Similar to Zhang<apos;>s work, Ni foam here behaves as both the substrate and the Ni precursor that was galvanically replaced by Cu(I) ion. Several other transition element-based MNs (A_1-x_M_x_N, A/M = Zn, Nb, Cr, Mo, V, Co, Ni, W, Ti and Fe etc.) were fabricated via this method [116,145–148].

Apart from NH_3_, other nitrogen sources have also been explored for the nitridation process. Notably, the pyrolysis of solid precursors is an effective method to generate porosity, further modulating surface microstructure and electronic structure. For example, Ojha and his coworkers successfully synthesized a hybrid nitride system composed of FeN_x_/Mo_2_N/C nanotubes by calcining mixed metal precursors and melamine under inert Ar flow for 10 h [149]. In another earlier work, Gomathi showed that mixed MNs (Fe_3_Mo_3_N, Co_3_Mo_3_N, and Ni_2_Mo_3_N) could be synthesized by heat treatment of their corresponding molybdate precursors (i.e. FeMoO_4_, CoMoO_4_, and NiMoO_4_) along with urea within a temperature range of 900 to 1000°C [150]. Yuan<apos;>s team systematically studied several MNs (MN_x_/C-uA, uA are in the oxidation of unsaturated alcohols, where M = Fe, Co, Cu, Cr, and Ni) synthesized at different pyrolysis temperatures using m-phenylenediamine as the nitrogen source; among them, iron nitride from metal salt (FeCl_3_ · 6H_2_O) presented the highest activity and selectivity to the corresponding aldehydes [151]. The catalysts prepared by thermal treatment at 900°C displayed better catalytic performance in selective oxidation with almost complete conversion and selectivity. It was further confirmed that the catalyst prepared at lower temperatures (~ 600°C) possesses a higher concentration of nitrogen-doped carbon that offered lower activity. In contrast, the formation of FeN_4_ was favored at higher synthesis temperatures, resulting in materials that offered better activity. Afterwards, this process was also utilized by Zhang et al., who adopted urea as an N source for Co- and N-doped VCoN nanoplates by a facile single-step pyrolysis process under Ar atmosphere (Figure 7c) [127]. As a result, superior catalytic activity (η_10_ = 179 mV) and long-term durability (100 h) in alkaline media were achieved.

Template-induced synthesis of mixed MNs is an effective method for generating tunable porous features that are crucial for their catalytic activity [152–154]. Fischer et al. developed a reactive hard templating approach for the fabrication of aluminium gallium nitride (AlGaN) and titanium vanadium nitride (TiVN) particles with diameters smaller than 10 nm, as demonstrated in Figure 7d [155]. Due to the confinement effect of the carbon nitride matrix, the composition of the resulting MN could be easily adjusted by changing the concentration of the preceding precursor solution. Thus, ternary MN nanoparticles with continuously tunable metal composition were successfully produced. Robins et al. presented ordered mixed titanium-niobium nitrides with gyroidal network structures synthesized from triblock terpolymer structure-directed mixed oxides [156]. The materials retained both macroscopic integrity and mesoscale ordering despite heat treatment up to 600°C without a rigid carbon framework as support. The gyroidal lattice parameters were varied by changing polymer molar mass. This kind of synthesis strategy may prove useful in generating a variety of monolithic ordered mesoporous mixed oxides and nitrides for electrode and catalyst materials.

Electrospinning and electrodeposition have been extensively used in the late 20th and early 21st centuries for the synthesis of a wide range of materials. Over the years, significant improvements have been made in the instrument design, the material used, and the nanomaterials synthesized [157,158]. In this context, Li et al. developed an electrospinning method combined with a post-nitridation treatment to fabricate 1D MNs at a large scale [159]. In a typical experiment, about 0.5 g nanofibers could be produced, and neither catalyst nor structural template was needed. A series of MNs, including TiN, VN, NbN nanofibers, and ternary MN nanofibers with controlled metal ratios, were prepared. In particular, unprecedented multi-channel TiN micro/nanotubes were also obtained. The number of channels could be readily controlled from one to three with specially designed electrospinning units, and the number of channels was directly associated with the surface areas of the samples. Zhang et al. fabricated 3D-NiMoN using a conventional two-electrode system in two steps on a carbon cloth substrate [160]. The NiMo alloy was synthesized using 30 mA/cm^2^ cathodic current density with 2 Hz frequency at ambient temperature. Afterwards, the alloy precursor was converted into NiMoN under 250 W nitrogen discharge plasma and 13.56 MHz frequency at 45°C for 15 min.

Magnetron sputtering deposition is a typical physical route for mixed MNs fabrication. In one representative work, bimetallic MoVN thin films were first deposited via magnetron sputtering deposition and were used as the HER electrocatalysts in an alkaline medium by Wei et al. [161]. In detail, MoVN thin film was sputtered on the conductive carbon paper under the Ar-N_2_ gases, and two high purity metal targets of Mo and V were employed as the metal precursors. Greczynski et al. reported a thin film synthesis technique that allows for unprecedented control over the crystalline phase formation in metastable transition MN-based layers [162,163]. A complete structural transition from hexagonal to the supersaturated cubic structure was achieved by tuning the incident energy of V_0.26_Al_0.74_N fabricated in Ar/N_2_ gas mixture. This finding enables the phase selective synthesis of novel metastable materials that combine excellent mechanical properties, thermal stability, and oxidation resistance. Chen et al. achieved highly crystallized ternary (Ti/Zr)N_x_ films by magnetron co-sputtering with different nitrogen gas flow ratios [164]. The structural and plasmonic properties of the films tuned by gas flow were investigated. All the films were solid solutions of TiN_x_ and ZrN_x_ with a rocksalt structure and preferred orientation. The films were nitrogen-over stoichiometric, and the main defects were cation vacancies, leading to the enhanced electronic density of states of nitrogen with increased nitrogen content. Therefore, elevating the energy level at which interband transition is excited gives a relatively high plasmonic quality in the visible and near-infrared region.

MBE or PLD technique is another physical vapor deposition technique that involves the vaporization of a source (target) through the action of a laser. The source is placed in a vacuum chamber or a chamber filled with specified gas (e.g. oxygen) while a high-power laser beam is directed. The target absorbs the energy, which subsequently excites its electrons [165]. Pau et al. [166] and Rodríguez et al. [167] successfully fabricated high-quality AlGaN and InGaN thin film with advanced photoresponse via MBE, respectively, indicating a bright future on epitaxial growth of mix-MNs. Tsuji et al. utilized an rf-plasma-assisted MBE system to represent a promising synthesis route for stabilized CaZn_2_N_2_ epitaxial films, as presented in Figure 7e [168]. By unintentional carrier doping, n- and p-type electronic conductions were attained with low carrier densities of the order of 10^13^ cm^−3^. Matenoglou et al. demonstrated the fabrication of 200–300 nm films of Ti_x_M_1-x_N and Ta_x_M_1-x_N on silica substrates [169]. The second harmonic Nd: YAG laser was used at room temperature (RT) in flowing N_2_ gas. Materials stoichiometric ratio ‘x’ changed with mixing Ti and Ta elements with suitable fixed metal targets in different ratios. Ti_x_Ta_1-x_N films formed solid solutions over the whole x range (0 < x < 1) and were stable in the rocksalt structure regardless of the valence electron configuration of the constituent metals. However, relatively few reports on MNs are based on the PLD technique until now, and further studies and development are urgently needed to further explore this direction.

Overall, mixed MNs are comprised of a huge class of compounds with a wide structural variety, rending them for a large range of applications. Common synthesis routes include thermal reduction, solid-state pyrolysis, high-temperature nitridation, physical deposition, and so on. Among them, physical synthesis strategies can produce materials with highly crystalline and better performance, while chemical methods are considered more flexible with a wider range of composition possibilities and large-scale production.

## 4. Synthesis of porous MNs

MNs exhibit versatile physico-chemical, catalytic, optical, and electronic properties owing to the presence of electronegative nitrogen atoms [170,171]. These materials find extensive application in the electrochemical fields such as energy storage [172]. As mentioned above, thermal annealing of the precursors, most often the metal oxides, is perhaps the most commonly utilized method for obtaining MNs [42]. One of the fascinating yet less explored aspects of MNs is the creation of porosity using various methods, which can improve their application efficacy as well as expand their application to more fields. For example, mesoporous molybdenum nitride with a high surface area of 121 m^2^ g^−1^ shows good efficiency for electron exchange due to its porous nature [173]. The introduction of porosity could be achieved through slight modifications of the process which involves either the use of sacrificial porous templates, the use of a molten salt approach, thermal annealing with/without modifications, or employing porous supports such as carbon, graphene or carbon nanotubes [174–176]. However, very few studies have investigated the porosity through the characterization of the textural features such as surface area, pore volume and pore size [177]. The forthcoming discussion has been designed to cover the synthesis of the porous MNs by providing comprehensive illustrations and comparative analysis. Table 3 summarizes some of the porous MNs and their various aspects of synthesis.

**Table 3.** Summary of the various aspects of the synthesis of various porous MNs

Material	Synthesis			Textural features		Ref.
	Precursors	Method	T (°C)	Surface area (m^2^ g^−1^)	Pore size (nm)	
Mo_2_N	Silica template, AMT	Silica templating/thermal annealing under NH_3_	800	121	8.6	[173]
Al-Ga-N, Ti-V-N	m-C_3_N_4_, GaCl_3_, AlCl_3_, TiCl_4_, VOCl_3_	Reactive template without NH_3_ usage	800	-	-	[155]
TiON	F127, (NH_2_)_2_CO, HCHO, Titanium oxo acetate	Self assembly and thermal annealing without NH_3_	800	24	9.5	[178]
VN, MoN, WN, TiN	Metal chloride, Li_3_N, ZnCl_2_	Thermal annealing without NH_3_	290	156.8, 124.5, 103.7, 135.2	5–75	[180]
(NSP-Co_3_FeN_x_/NF)	Metal double hydroxide, Ni foam	Hydrothermal cum thermal annealing under NH_3_	350	62.77	-	[41]
CoN, Fe_4_N, Ni_3_N	Metal inks, Ni foam	Thermal annealing under NH_3_	500	-	-	[45]
Fe_3_N/Fe_4_N	Graphene, Ni foam, Fe(NO_3_)_3_	Thermal annealing under NH_3_	400	-	-	[183]
ZFN-900	Fe(NO_3_)_3_.9H_2_O, Zn(NO_3_)_2_.6H_2_O ZIF-8,	Thermal annealing under ammonia	900	1027	2.3	[184]
VN	VCl4, (NH_2_)_2_CO	Stirring and ultrasonication	70	144.43	4.68	[43]

**Note: T** – Temperature, Mo_2_N – Mesoporous molybdenum nitride, AMT – Ammonium molybdate tetrahydrate, Al-Ga-N – Aluminium Gallium nitride, Ti-V-N, Titanium vanadium nitride, m-C_3_N_4_ – mesoporous carbon nitride, GaCl_3_ – Gallium chloride, AlCl_3_ – Aluminium chloride, TiCl_4_ – Titanium chloride, VOCl_3_ -Vanadium oxychloride, TiON – Titanium oxy nitride, F127 – Pluronic tri block copolymer, (NH_2_)_2_CO – Urea, HCHO – Formaldehyde, VN – Vanadium nitride, MoN – Molybdenum nitride, WN – Tungsten nitride, TiN – Titanium nitride, Li_3_N – Lithium nitride, ZnCl_2_ – Zinc Chloride, NSP-Co_3_FeN_x_/NF – Nanoparticle stacked porous cobalt nitride nanowires on nickel foam, CoN, Fe_4_N, Ni_3_N – Cobalt nitride, iron nitride and nickel nitride on Nickel foam, Fe_3_N/Fe_4_N – Nanoporous iron nitride film grown on 3D graphene/Ni foam as a porous support, ZFN-1000 – Fe_3_N encapsulated within porous carbon derived from ZIF-8, Fe(NO_3_)_3_.9H_2_O – Iron nitrate, Zn(NO_3_)_2_.9H_2_O – Zinc nitrate, ZIF-8 – Zeolitic imidazole framework, VN – Mesoporous vanadium nitride, VCl_4_ – Vanadium tetrachloride,

### 4.1 Reactive templating

Reactive templating using mesoporous carbon nitride is one of the facile approaches to preparing binary or ternary nitrides. Mesoporous carbon nitride provides the dual effect of acting as a replica template as well as a reactive source of nitrogen. For example, an earlier report suggested the synthesis of two types of ternary MNs (Al-Ga-N and Ti-V-N) with particle size< 10 nm using mesoporous graphitic C_3_N_4_ as a reactive template [155]. It is anticipated that the mesoporosity of the carbon nitride would have replicated in the final materials, however, the characterization of textural features such as surface area, pore volume and pore size was missing. The synthesis of the ternary MNs was accomplished via the following steps; 1) synthesis of mesoporous carbon nitride (C_3_N_4_), 2) preparation of ethanolic solutions of metal chlorides, 3) mixing, sonication and forced evaporation of mesoporous C_3_N_4_ and solution of metal chlorides, and 4) heat treatment. The use of carbon nitride as a reactive template is an effective ammonia-free method to generate porous MNs, however, more research into avoiding oxygen in the final materials by using suitable starting metal salts is one of the top priorities to be addressed.

### 4.2 Self-assembly and thermal annealing

**Figure 8.**
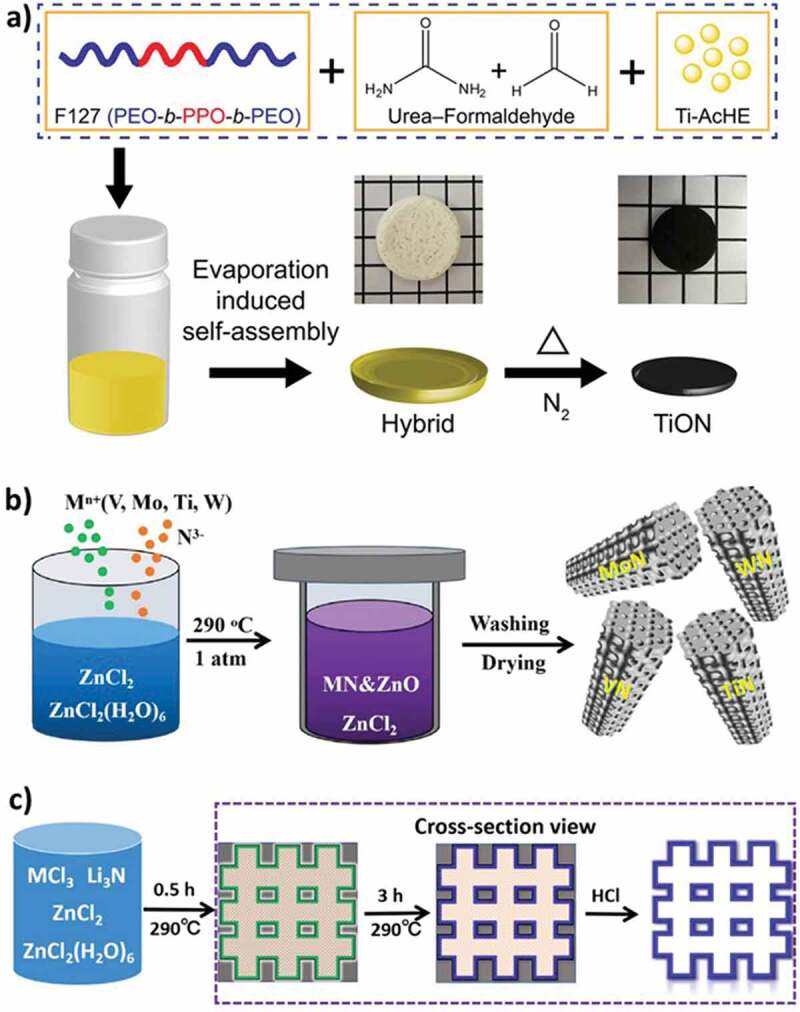
**A)** Synthesis scheme of crystalline mesoporous titanium oxynitride (Reproduced with permission from [178]) **b)** synthesis scheme of porous single MNs of vanadium, molybdenum, titanium, and tungsten via molten salt route, and **c)** their formation mechanism (Reproduced with permission from [180]).

The combined approach of self-assembly of the structure-directing agent with the metal and nitrogen precursors followed by thermal treatment is an effective way of introducing porosity in the MNs. For the self-assembly approach, unlike carbon nitride that is mesoporous and acts as a reactive template, an external block copolymer is required to direct the structure and induce porous structure in the synthesized MN. For example, heat treatment of a combined mixture of Pluronic F127 triblock copolymer, urea-formaldehyde and metal precursor titanium-oxo-acetate at 800°C produced ordered mesoporous monoliths of crystalline titanium oxynitride with an average pore diameter of 9.5 nm and surface area of 24 m^2^ g^−1^ (Figure 8a) [178]. Although the surface area of the materials is not high, this combination of block copolymer directed self-assembly of the reaction compounds followed by thermal annealing is indeed a successful strategy for the conversion of metal precursors into metal oxynitrides. The synthesis of highly pure MNs may require the careful choice of starting precursors containing little to no oxygen such as metal chlorides to avoid the incorporation of nitrogen into the structure. Urea can also be combined directly with metal oxide salts at high temperatures to yield MNs without the use of ammonia, however, the lack of porosity could hinder the application performance of such materials [150].

### 4.3 Molten salt route

Molten salt route is one of the viable methods to generate porosity as well as reduce the overall length of the synthesis procedure. It has been very well demonstrated for the synthesis of porous carbon-based materials with zinc chloride being the most popular reagent for the purpose [179]. The molten salt route can be extended to synthesize porous MN as well. For example, vanadium, molybdenum, tungsten, and titanium nitrides with a reasonable high surface area can be synthesized from their respective chloride salts by activation with zinc chloride [180]. A mixture of individual metal chlorides, lithium nitride (Li_3_N), and zinc chloride in both hydrated and anhydrous forms was grounded into a solid mass and subjected to heating at a relatively low temperature of 290°C to obtain porous MNs (Figure 8b). This method is highly attractive in terms of avoiding the use of toxic ammonia for nitridation, quicker synthesis, avoiding oxygen in the final materials and most significantly a low temperature for the synthesis. It was proposed that the formation of porous MNs takes place through the deposition of metal and nitrogen on 3D Zinc oxide (ZnO) formed during the synthesis at 0.5 and 3 h intervals and the subsequent washing of ZnO with acid yields the crystalline porous MN as the final material (Figure 8c). The surface area of VN (156.8 m^2^ g^−1^), MoN (124.5 m^2^ g^−1^), WN (103.7 m^2^ g^−1^), and TiN (135.2 m^2^ g^−1^), their respective pore volumes (0.81, 0.52, 0.26, 0.89 cm^3^ g^−1^) and pore size in the range of large-sized mesopores and macropores suggest that the materials are porous and could be suitable for different applications.

### 4.4 Ammonia thermal annealing

**Figure 9.**
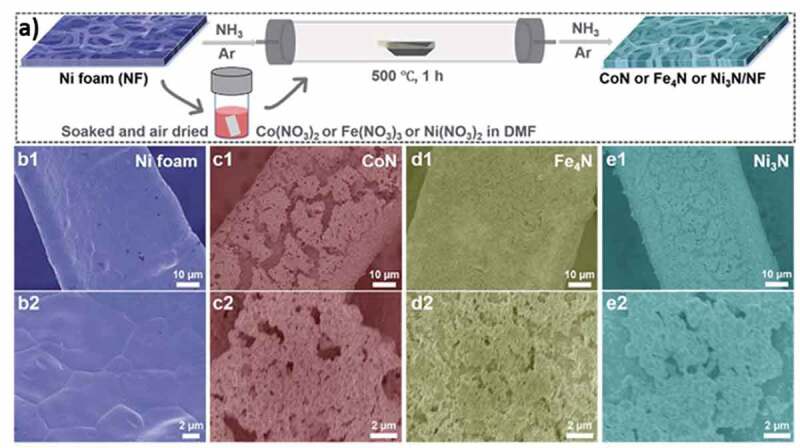
**A) S**ynthesis scheme for MNs of cobalt, iron and nickel, and the SEM images of **b1-b2)** nickel foam, **c1-c2)** cobalt nitride, **d1-d2)** Fe_4_N, and **e1-e2)** Ni_3_N (Reproduced with permission from [45]).

**Figure 10.**
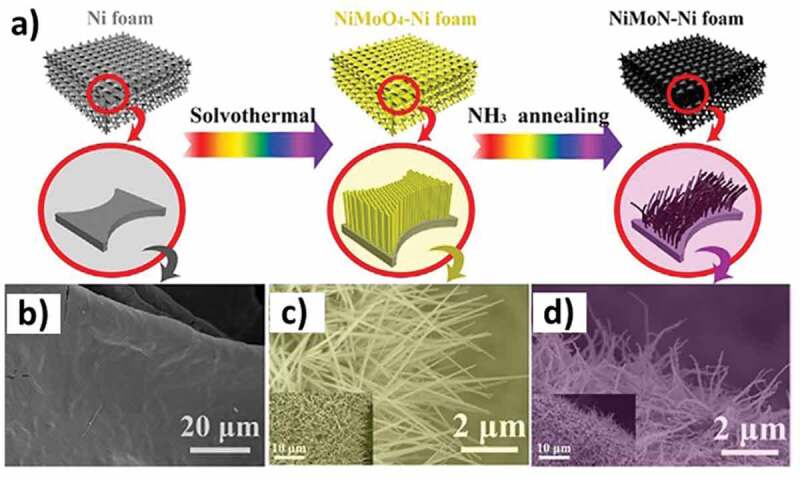
**A)** Synthesis scheme, and **b-d)** SEM images of the nickel molybdenum nanowires (Reproduced with permission from [181]).

Ammonia thermal annealing using ammonia to incorporate nitrogen in metals is a widely reported method; however, the environmental concerns with the use of ammonia push back any commercial plan for the synthesis of materials on a bulk scale. Moreover, the porosity achieved in such an MN is not that high as compared to porous MNs prepared using other methods. For example, a mixed MN composed of cobalt and iron synthesized using ammonia treatment showed a surface area of 62.77 m^2^ g^−1,^ which is comparable to the materials discussed in some of the previous examples [41]. During the synthesis, the mixed metal double hydroxide (CO_3_FeDH) was grown on a nickel foam (CO_3_FeDH/NF) followed by ammonia-based thermal annealing at 350°C to obtain nanoparticle stacked porous cobalt nitride nanowires (NSP-CO_3_FeN_x_/NF). The porous nature of the nanowires and their high electrical conductivity made them facile candidates for the overall water splitting. A similar approach was also employed for the synthesis of porous monometallic (CoN, Fe_4_N, Ni_3_N) and porous bimetallic nitrides (NiFeN, CoFeN, NiCoN) [45]. To synthesize monometallic nitrides, the individual metal precursors in the form of ink were soaked with Ni foam and the dried mixture was thermally annealed under NH_3_ at 500°C. The metal inks were mixed to realize the mixed nitrides (Figure 9a). The porosity of the materials was not evaluated using nitrogen sorption, however, the SEM images identified the conversion of the smooth surface of the nickel foam with MN-based nanoparticles (Figure 9b). Another similar instance reports the synthesis of NiMoN nanowires achieved through the solvothermal combination of nickel foam with ammonium molybdate and nickel nitrate salts followed by thermal annealing under NH_3_ at 700°C (Figure 10a) [181]. The nanowires grew on nickel foam, which was evident from the SEM images (Figure 10b). The nanostructured morphology in the form of nanowires was beneficial for the materials and it displayed good performance as an electrocatalyst for overall water splitting.

### 4.5 Thermal annealing with porous support

**Figure 11.**
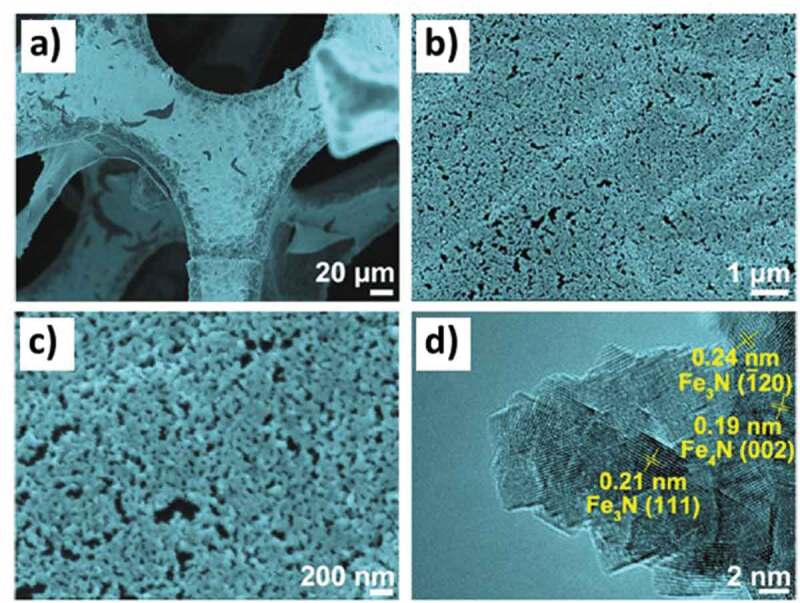
**A-c)** SEM, and **d)** TEM image/s of the nanoporous iron nitride supported on 3D graphene/nickel foam (Reproduced with permission from [183]).

**Figure 12.**
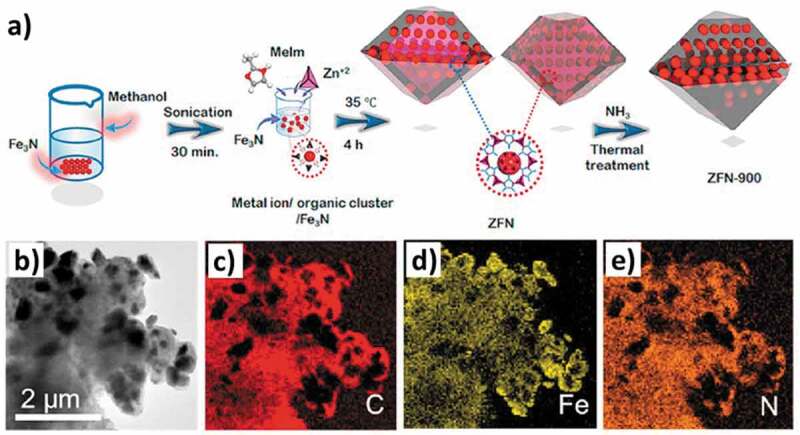
**A)** Synthesis scheme, **b)** TEM image and **c-e)** EDS mapping results for Fe_3_N encapsulated inside ZIF-8 derived porous carbon (Reproduced with permission from [184]).

Inducing porosity in materials by supporting them on porous support is a fairly common procedure and it has been applied for MNs as well [182]. Thermal annealing is a critical component of the process for establishing a connection between the support and the material. For example, an iron nitride (Fe_x_N) film supported on a three-dimensional graphene/nickel foam was synthesized through the dispersion of the two components followed by ammonia-based thermal annealing at 400°C [183]. The authors did not report on the characterization of the textural features using nitrogen sorption; however, the microscopic examination gave clear evidence of the nanoporous nature of the synthesized MN film on the porous support (Figure 11a-d). The characterization using various data revealed that the iron nitride film grows in two compositions, Fe_3_N and Fe_4_N on the porous support and is highly crystalline. The nanoporosity of the material favored a fast charge transfer in oxygen evolution reaction (OER), which occurs due to the availability of a higher number of active catalytic sites. Iron nitride with a composition of Fe_3_N is suitable for electrochemical purposes. One of the facile methods to increase the application efficiency of Fe_3_N is to encapsulate it in a porous carbon as a support and the composite exhibits a high surface area of 1027 m^2^ g^−1^ with a pore size of 2.3 nm [184]. The synthesis proceeds with the formation of Fe_3_N from Fe_2_O_3_ at 900°C under NH_3_ atmosphere, which is then encapsulated inside ZIF-8 via sonication in methanol to obtain ZFN (Figure 12). The thermal annealing of ZFN at a temperature range of 800–1000°C under NH_3_ atmosphere produces final materials with high stability, methanol tolerance and uniform distribution of Fe and N in carbon matrix (Figure 12b **and c-**e). Similarly, molybdenum nitride (Mo_2_N) nanoparticles could be dispersed in nitrogen doped carbon nanotubes to obtain a material with a reasonable high surface area of 369 m^2^ g^−1^ and pore size of ~6 nm [185]. Nitrogen doped graphene nanosheets is another precursor for loading molybdenum nitride [186].

Overall, structural modification in MNs is one of the effective strategies to enhance their application efficacy [187,188]. Introducing porosity in MN is not a straightforward operation, however, if present, the porosity becomes a crucial factor for enhancing the electrochemical performance of MNs. To date, not much literature has under-addressed the porosity in MNs which have limited their application. Recent studies on generating porosity in MNs using reactive templates such as carbon nitrides, porous supports, molten salt routes have yielded encouraging results in the terms of the material structure and properties and also form the point of view of enhancement in application performance. Therefore, designing novel methods for creating a new generation of porous MNs should become the research forefront in the subject.

## 5. Electrochemical production of hydrogen

MN-based materials can be a viable option for the replacement of conventional noble metals for electrochemical hydrogen production due to their excellent properties such as the availability of abundant electrochemical active sites, high electronic conductivity, and thermal and mechanical stability, and tunable surface and morphology [114]. In particular, the introduction of the interstitial nitrogen atoms results in increased lattice distance of the metal which imparts a noble metal like electronic structure with excellent electron donating ability and high catalytic activity for HER [189]. There are main parameters that are needed to understand the electrocatalytic activity for HER. These include the overpotential, Tafel slope, electrochemical active surface area, pH of solution, turnover frequency and Gibbs-free energy [190]. The structure of MN can be optimized by doping, making hybrids/composites and introducing porosity which are beneficial for enhancing the HER activity of the catalyst. In this section, the various MNs and their performance with respect to their reaction kinetics of HER activity from experimental results and density functional theory (DFT) calculations are discussed and a brief summary is also provided in Table 4.

**Table 4.** MN-based materials as catalyst for HER

Material Type	Catalyst	Electrolyte	Overpotential (mV) at 10 mA cm^−2^	Tafel Slope (mV/dec)	Stability	Reference
Mo	FeN_x_/Mo_2_N/CNTs	0.5 M H_2_SO_4_	180	166	42 h	[149]
	2D C_3_N_4_@MoN	1.0 M KOH	110	57.8	10 h	[191]
	MoS_2_–Mo_2_N	0.5 M H_2_SO_4_	121	49.6	20 h	[192]
	MoP/MoN_2_	1.0 M KOH	89	78	3000 cycles	[203]
Ni	Ni_3_FeN-nanoparticles	1.0 M KOH	238	46	-	[205]
	IrNiN/C	0.1 M HClO_4_	-	36	-	[204]
	Ni_3_N@VN–NF	1.0 M KOH	56	47	2000 cycles	[206]
	Co_2_Ni_1_N	0.5 M H_2_SO_4_	92	55.3	1000 cycles	[211]
Co	Co-Mo-N@Ag	1.0 M KOH	90	73	30 h	[212]
	CeO_2_/Co_4_N	0.5 M H_2_SO_4_	33	65	12 h	[215]
	CoN-400	1.0 M KOH	97	93.9	35 h	[218]
W	P-WN/rGO	0.5 M H_2_SO_4_	85	54	5000 cycles	[219]
	2D-W_2_N_3_	0.5 M H_2_SO_4_	98.2	59	1000 cycles	[224]
	WN nanowire	1.0 M KOH	130	57.1	3000 cycles	[36]
V	Co, N-codoped porous vanadium nitride	1.0 M KOH	179	123	100 h	[226]
	Ru-VN-2	0.5 M H_2_SO_4_	134	35	20 h	[225]
	Co-VN-nanosheet array	1.0 M KOH	37	41	2000 cycles	[36]
Ti	Cu(20)@3DOM-TiO_2_	1.0 M KOH	71	51	1000 cycles	[229]
	TiN/MoS_2_	0.5 M H_2_SO_4_	146	44.8	-	[230]
	NiFeP-TiN	1.0 M KOH	75	73	10 h	[36]
Fe	CoAl-Fe_2_N/Fe_3_N	1.0 M KOH	54	145	10 h	[235]
	Ni_3_FeN/r-GO	1.0 M KOH	94	90	10 h	[210]
	FeNi-N	1.0 M KOH	106	115	25 h	[236]
Cu	Cu_3_N@CoNi carbonate hydroxides	1.0 M KOH	182	134	24 h	[238]
	Cu_x_Ni_4-x_N	0.1 M KOH	58	67	500 cycles	[237]
	Cu_1_Ni_2_-N	1.0 M KOH	71.4	106.5	60 h	[236]
Nb	Nb-Ti Nitride Nanotube Array	1.0 M KOH	120	52.9	100 h	[232]
	Nanoporous Nb_2_N	0.5 M H_2_SO_4_	96.3	92	20 h	[241]
Ga	Pt/Porous GaN	1 mM H_2_PtCl_6_/0.5 M NaCl	98	85	1000 cycles	[243]
	GaN(100)	0.5 M H_2_SO_4_	168	36	10 days	[244]

**Note**: FeN_x_/Mo_2_N/CNTs – Iron nitride/Molybdenum nitride/N-doped carbon nanotube composite, 2D C_3_N_4_@MoN – 2D graphitic carbon nitride/molybdenum nitride hybrid, MoS_2_–Mo_2_N – molybdenum disulfide/molybdenum nitride, MoP/MoN_2 –_ Porous Molybdenum Phosphide/Nitride, Ni_3_FeN-nanoparticles – Iron–Nickel Nitride Nanoparticles, IrNiN/C – iridium–nickel nitride nanoparticles on carbon cloth, Ni_3_N@VN–NF – Nickel nitride@vanadium nitride on nickel foam, Co_2_Ni_1_N – Porous Cobalt/Nickel Nitride, Co-Mo-N@Ag – Silver nanoparticles decorated cobalt molybdenum nitride, CeO_2_/Co_4_N – oxygen vacancy-rich cerium oxide supported on cobalt nitride, CoN-400 – cobalt nitride porous nanowires on carbon cloth, P-WN/rGO – Phosphorus-Modified Tungsten Nitride/Reduced Graphene Oxide, 2D-W_2_N_3 –_ 2D Nitrogen-Rich Tungsten Nitride, WN nanowire – tungsten nitride nanowire array on carbon cloth, Ru-VN-2 – Ruthenium doped vanadium nitride, Co-VN-nanosheet array – Cobalt doped vanadium nitride nanosheet arrays, Cu_3_N(20)@3DOM-TiO_2 –_ 3D ordered macroporous copper nitride–titanium oxynitride, TiN/MoS_2 –_ Molybdenum sulfide nanosheets on titanium nitride nanorods, NiFeP-TiN – Ni-doped amorphous iron phosphide nanoparticles on titanium nitride nanowire arrays, CoAl-Fe_2_N/Fe_3_N – Cobalt and aluminium doped iron nitride, Ni_3_FeN/r-GO – Nickel iron nitride nanoparticles on reduced graphene oxide aerogels, FeNi-N – Porous Nickel-Iron Nitride Nanosheets, Cu_3_N@CoNi carbonate hydroxides – copper nitride and Cobalt nickel carbonate hydroxides on copper foam, Cu_x_Ni_4-x_N – nickel-copper nitride, Cu_1_Ni_2_-N – Copper–Nickel Nitride Nanosheets, Re-Ni/NNA – niobium and titanium nitride nanotube arrays implanted with nanosized amorphous rhenium nickel, Nanoporous Nb_2_N – Nanoporous niobium nitride, Pt/Porous GaN – platinum nanoparticles on porous gallium nitride, GaN(100) – Gallium Nitride Single-Crystalline Facets

Various nitride-based materials with metals including Mo, Ni, Co, W, V, Ti, Fe, Cu, Ga, and Nb are commonly used as the active catalysts for HER. Among all these materials, Mo-based nitrides are widely studied for HER with enormous number of reports on related hybrid materials including carbon nanotubes (CNTs) [149], C_3_N_4_ [191], metal sulfides [192,193], metal oxides [194,195], metal carbides [196,197], bimetallic [198,199], heteroatom doped [200], and metal doped [201,202]. Jin et al. developed heterostructured 2D C_3_N_4_@MoN for an excellent HER activity with a small Tafel slope of 57.8 mV/dec. It was found that the enhanced interfacial interaction of C_3_N_4_ and MoN heterostructures and the unique electronic structure are responsible for the high HER activity [191]. This is also well matched with the DFT calculations which show optimized (−0.23 eV) hydrogen adsorption-free energy (ΔG_H*_) of the C_3_N_4_@MoN composite at N (C_3_N_4_) sites for favorable HER activity when compared to g-C_3_N_4_ and MoN. At the same time, the hydroxyl adsorption-free energy ΔG_OH*_ at the Mo site of C_3_N_4_@MoN exhibits relatively negative value of −0.45 eV when compared to g-C_3_N_4_ and MoN and promotes stronger OH* adsorption for enhanced HER kinetics in the alkaline electrolyte used. Using carbon cloth as a substrate, Huang et al. developed MoS_2_–Mo_2_N hybrid heterostructure, which showed an overpotential of 121 mV @ 10 mA cm^−2^ and Tafel slope of 49.6 mV/dec. In this hybrid system, the charge transfer was much better than that of Mo_2_N and MoS_2_ [192]. Here, the 3D carbon cloth and Mo_2_N greatly prevent the disintegration of the MoS_2_ and thereby enhance the chemical stability of the composite by retaining 97.4% at 50 mA cm^−2^ for 20 h. In another report, Ojha et al. demonstrated the HER activity of FeN_x_/Mo_2_N/CNTs which offered a Tafel slope as low as 166 mV/dec and small overpotentials of 180, 218, and 400 mV at 10, 20, and 100 mA cm^−2^ by optimizing the iron salt [149]. Here, the introduction of N-doped CNT to the bimetallic nitride not only increases the electrical conductivity but also enhances the charge transfer which significantly improved the HER activity with a high electrochemical stability for 42 h. In another report, Gu et al. studied the performance of porous and non-porous MoP/MoN_2_ catalyst for HER in KOH, phosphate buffer solution (PBS) and H_2_SO_4_ solution [203]. Regardless of the electrolyte used, the 2D porous MoP/MoN_2_ exhibited better HER activity than non-porous MoP/MoN_2_ which is linked with the large turnover frequency (0.06 s^−1^) and large electrochemical active surface area (85.0 mF cm^−2^) of porous MoP/MoN_2._ It is noteworthy to mention that this porous Mo-based nitride/phosphide heterojunction material with optimized H* adsorption even registered higher HER activity than Pt/C at a current density of >55 mA cm^−2^ and >190 mA cm^−2^ in neutral and alkaline medium, respectively.

**Figure 13.**
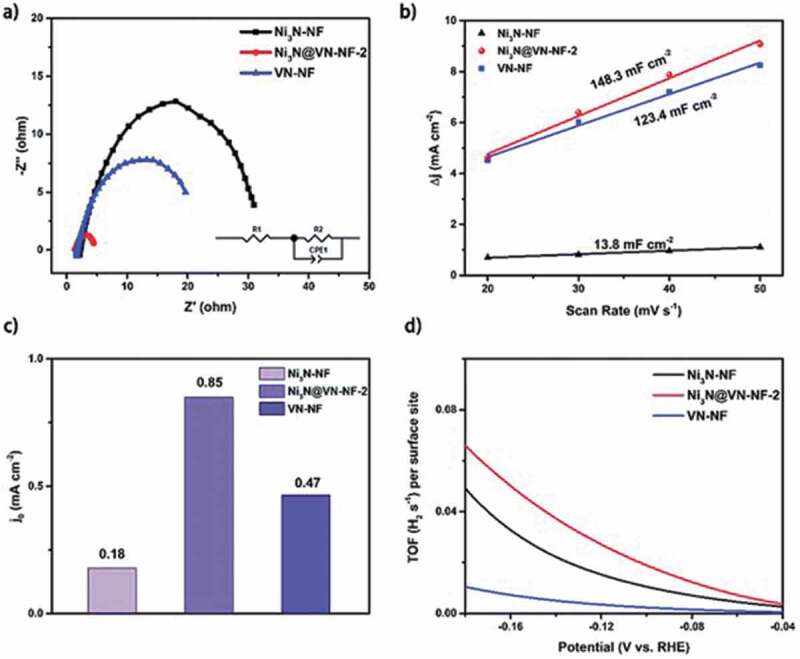
Comparison of electrodes Ni_3_N–NF, VN–NF and Ni_3_N@VN–NF-2 for HER activity with respect to (a) Electrochemical impedance spectroscopy (b) Electrochemical surface area using electrochemical double layer capacitance (c) Exchange current density (d) Turnover frequency (Reproduced with permission from [206]).

Next to Mo, substantial work has also been done on the functionalized Ni nitride-based materials [37,204–210], and their HER activity. For example, Chen et al. demonstrated that Ni_3_FeN nanoparticles with Brunauer–Emmett–Teller (BET) surface area of 15.4 m^2^/g can outperform NiFe-layered double hydroxide (BET surface area of 10.2 m^2^/g) when utilized as a cathode for HER evident from smaller Tafel slope and overpotential [205]. More importantly, unlike the conventional method of using powdered electrocatalyst, the carbon cloth as a substrate can offer enhanced electrical conductivity, better mechanical properties, and smooth charge transfer from 3D nanostructures for fast kinetics of HER activity. In a water electrolyzer, the Ni_3_FeN nanoparticles showed high stability for more than 130 h. Kuttiyiel et al. used a unique technique for enhancing the HER activity of MNs. In one of the approaches, they demonstrated that coupling Ni nitrides with Ir Ni cores can significantly enhance the HER activity. The prepared core-shell iridium nickel nitride registered a better Tafel slope and current exchange density for IrNiN/C(36 mV/dec, 0.613 mA cm^−2^) when compared to Ir/C(59 mV/dec,0.452 mA cm^−2^) and Ni/C(168.3 mV/dec, 3.52 × 10^–4^ mA cm^−2^) [204]. This HER activity is close to Pt/C (Tafel slope of 30.4 mV/dec) where the nitridation of Ir and Ni brings contradiction of d band centre of Ir and a relatively lesser hydrogen binding energy for improved HER activity. To increase the conductivity of the MNs, Zhou et al. introduced highly conductive VN and designed Ni_3_N@VN-NF cathode with a BET surface area of 53.1 m^2^/g, showing a better Tafel slope (47 mV/dec) and small overpotential (56 mV) in 1 M KOH solution when compared to Ni_3_N-NF and VN-NF [206]. The parameters such as electrochemical impedance spectroscopy, electrochemical double-layer capacitance, exchange current density and turn over frequency greatly determine the HER activity and hence the comparison is studied as in Figure 13. MOF-74 was used as a starting material to prepare porous Co_3_N, Ni_3_N and Co_2_Ni_1_N and tested on different pH solutions as electrolytes [211]. Although the BET surface area of Ni_3_N (250.7 m^2^/g) is higher than Co_2_Ni_1_N (244.4 m^2^/g), the electrochemical surface area of the bimetallic nitride (19.16 mF cm^−2^) plays a major role in enhanced performance when compared to Ni_3_N (8.77 mF cm^−2^) and Co_3_N (11.5 mF cm^−2^) with Tafel slope as low as 55.3 mV/dec in 0.5 M H_2_SO_4_.

**Figure 14.**
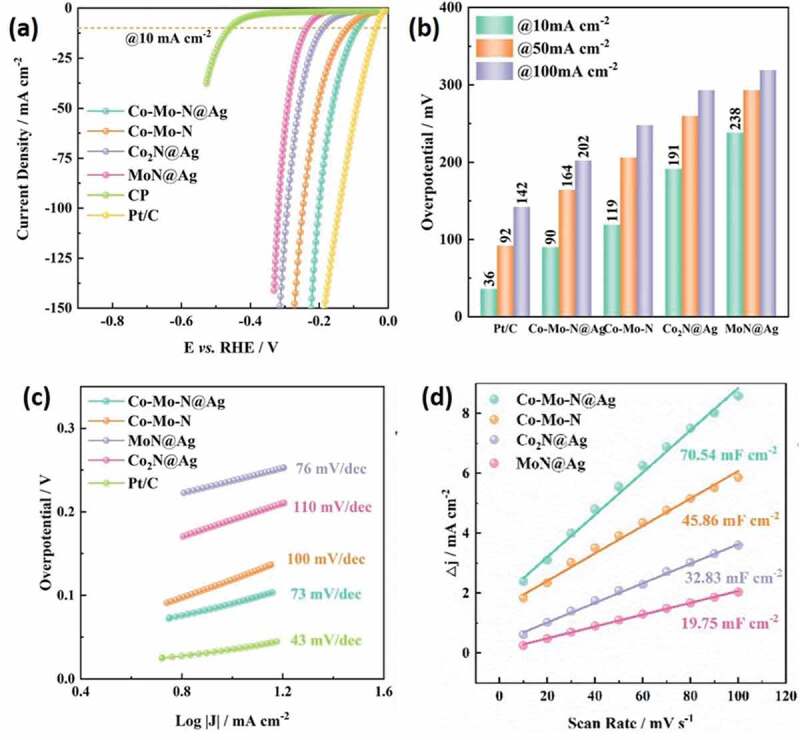
Comparison of HER catalyst materials Co-Mo-N@Ag, Co-Mo-N, Co_2_N@Ag, MoN@Ag, and Pt/C (a) Polarization curves of HER (b) Overpotential at different current densities (c) HER Tafel plots (d) HER Electric double layer capacitance (C_dl_) (Reproduced with permission from [212]).

Cobalt-based nitrides and their composites with other nitrides [212,213], metal oxides [214,215], metal phosphides [216], and metal sulfides [217] were considered as effective HER electrocatalysts. Using electrodeposition technique, Ag-doped Co-Mo nitride were prepared and found to register smaller charge transfer resistance (R_CT_) and overpotential (4.5 Ω, 90 mV) at 10 mA cm^−2^ when compared to Co_2_N@Ag(57.9 Ω, 191 mV), MoN@Ag(13.9 Ω, 238 mV), and Co-Mo-N(21.18 Ω, 119 mV), showcasing the effect of additional catalytic sites from bimetallic nitride and optimized electronic structure from Ag doping (Figure 14) [212]. Yao et al. used CeO_2_ to boost the HER performance of Co_4_N by increasing the surface area, active catalytic sites and surface defects. As expected, when H_2_SO_4_ is used as an electrolyte, the Tafel slop and overpotential improve significantly from 110 to 65 mV/dec and 165 to 33 mV for Co_4_N and CeO_2_/Co_4_N. These results are also correlated with the smaller resistance from the Nyquist plot and larger double-layer capacitance for CeO_2_/Co_4_N (79.2 mF cm^−2^) when compared to Co_4_N (6.7 mF cm^−2^). A binder-free approach is used with carbon cloth as a substrate for the preparation of porous CoN nanowires for HER activity [218]. The catalyst and substrate enable high conductivity with enhanced charge transport kinetics, high stability (35 h) and low overpotential of 97 mV which is much better when compared to Co_3_O_4_ (229 mV).

Tungsten-based nitrides and their combinations with reduced graphene oxide (rGO) [219], bimetallic nitrides [220], metal doping [221], metal carbides [222], and metal hydroxides [223] have been extensively used as electrocatalysts for HER. For example, Yan et al. demonstrated that even a small amount (2.52 at%) of phosphorus doping can greatly enhance the HER activity of WN/rGO as it significantly alters the electronic state and increases the electron density and the active sites of the material [219]. Further, the optimized material with P doping was able to retain 92% of initial current density at an overpotential of 120 mV and 20 h test, which is much higher when compared to undoped catalyst (80%). To this end, Yu et al. prepared 2D W_2_N_3_ which is rich in nitrogen and acting as an effective candidate for HER activity [224]. On comparison with bulk W_2_N_3_, this newly prepared material has smaller overpotential (98.2 mV) and better Tafel slope (59 mV/dec), resulting from the abundant active sites from the favorable 2D structure. Porosity is also introduced to enhance the HER activity of the MNs. For example, porous WN nanowires with a BET surface area of 3.98 m^2^/g is developed by plasma nitridation of WO_x_ and tested for HER activity [36]. This material exhibits a low Tafel slope of 57.1 mV/dec with a high current exchange density of 6.6 × 10^–2^ mA cm^−2^ and a low charge transfer resistance of 4.6 Ω, revealing its high electron transport capability.

Vanadium nitride-based materials and their hybrids with metals [199,225–227], and metal sulfides [228] have been used as electrocatalysts for HER applications. For example, Zhang et al. demonstrated the preparation of Co, N-codoped porous vanadium nitride with a high BET surface area (21.4 m^2^/g) and presented its excellent HER activity. The prepared materials showed a small overpotential of 179 mV at 10 mA cm^−2^ and a Tafel slope of 123 mV/dec [226]. Notably, the pyrrolic and graphitic N sites and Co doping bring additional active sites and improve conductivity while the material is highly stable for up to 100 h. In another work by Wang et al., Ru doped VN was used as an effective HER catalyst where the optimized material Ru-VN-2 showed excellent Tafel slop of 35 mV/dec and a small overpotential of 134 mV at 10 mAcm^−2^ in 0.5 M H_2_SO_4_ solution [225]. Further, for the optimized Ru doping, a large turnover frequency of 1.77 s^−1^ at 200 mV and stability for 20 h without much degradation is achieved, showing the importance and effect of Ru doping. By simple doping of Co to VN nanosheet array samples, the HER activity can be significantly enhanced. For example, Co-doped VN registered a small overpotential of 37 mV and a Tafel slope of 41 mV/dec which is close to those of Pt/C [227]. It was demonstrated that the high electronic conductivity, high BET surface area (26.5 m^2^/g), high electrochemical double-layer capacitance (40.3 mF cm^−2^) and optimized hydrogen adsorption Gibbs-free energy facilitates a rapid electron transport and enhance HER performance in comparison to VN nanosheet array.

Another material that has proved to be a potential HER cathode is Ti nitride. Various TiN nanostructures with oxynitrides [229], other metals [208], metal sulfide [230], metal oxide [231], bimetallic [232], and porous [233] have been explored. For example, Wu and co-workers studied the performance of copper nitride/3D porous titanium oxynitride as an HER catalyst in alkaline, acidic, and neutral media [229]. Both Tafel slop and linear sweep voltammetry (LSV) measurements show that the material performed best in 1 M KOH followed by 1 M PBS and 0.5 M H_2_SO_4_ for HER. Moreover, the DFT calculations demonstrated that the activation energy of the Cu_3_N (100) surface is 0.519 eV which is much lower compared to Cu (100) surface (0.924 eV) and hence the nitridation plays a major role in improving the kinetics of HER activity. The 3D porous structure of titanium oxynitride with the large BET surface area (168 m^2^/g) and electrochemically active surface area (1.43 mF cm^−2^) contributed to enhanced surface mass transport and fast kinetics for hydrogen production. Yu et al. performed the catalytic studies on MoS_2_ nanosheets on TiN nanorods and observed large improvement in Tafel slope (65.6 to 44.8 mV/dec) and overpotential at 10 mA cm^−2^ (252 to 146 mV) when compared to pristine MoS_2_ [230]. It was demonstrated that the enhanced activity is due to the synergistic effect of fast electron kinetics from highly conductive TiN nanorods and smooth ion diffusion from MoS_2_ nanosheets. Peng et al. prepared Ni-doped FeP nanoparticles decorated on TiN nanotube arrays with carbon cloth as substrate possessing high electrical conductivity and fast electron transport exhibiting Tafel slope as low [234] as 73 mV/dec. The Ni doping enhances the electrochemical active surface area by 18% while the overpotential decreases from 176 mV (FeP-TiN) to 75 mV (Ni-FeP-TiN) as the doping finely tune the electronic structure and the orbital orientation which are critical for the enhancement of the electrocatalytic activity.

**Figure 15.**
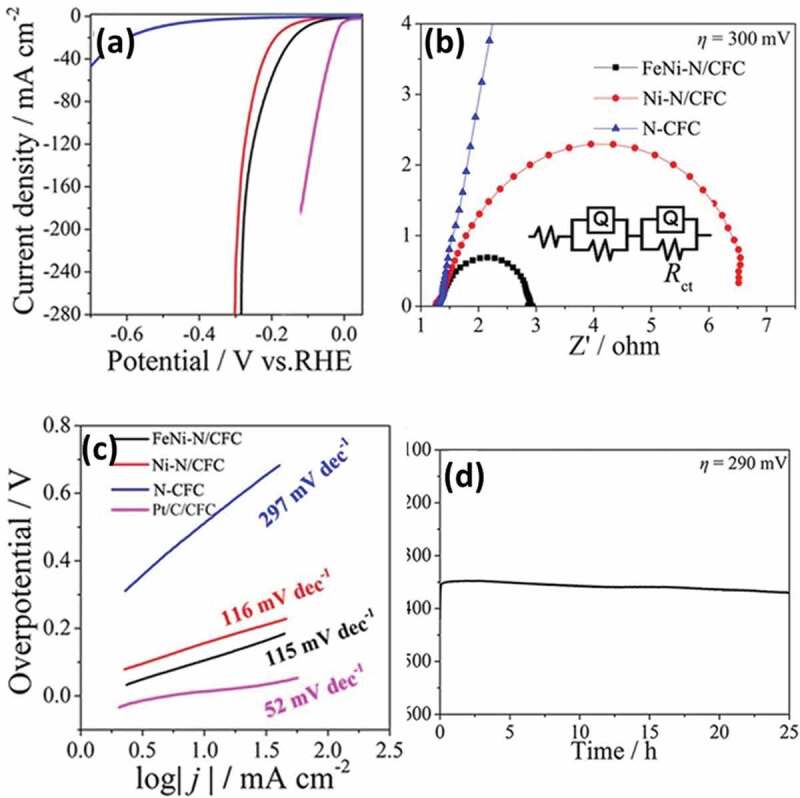
Comparison of different materials FeNi-N/CFC, Ni-N/CFC, N-CFC and Pt/C on (a)HER polarization curves (d) Nyquist plot and equivalent circuit (c) HER Tafel slope (d) Study of current density curve with respect to time for FeNi-N/CFC Reproduced with permission from [236]).

Similarly, iron nitride-based materials are also demonstrated as the excellent catalysts for HER [149,205,210,235]. Hu et al. reported a simple doping strategy for the preparation of CoAl-Fe_2_N/Fe_3_N which not only tuned the d band centre of iron nitride but also offered favorable interface engineering on Fe_2_N/Fe_3_N [235]. As expected, the doped metallic nitride delivered a Tafel slope and overpotential of 54 mV/dec and 145 mV, which is much smaller when compared to pristine Fe_2_N (461 mV, 89 mV/dec). Notably, Gu et al. developed Ni_3_FeN as a bimetallic catalyst and also studied the effect on the making composite with r-GO aerogel for HER activity in KOH showing a Tafel slope of 90 mV/dec [210]. It was demonstrated that the incorporation of r-GO onto Ni_3_FeN offers improved conductivity and additional catalytic active sites which are responsible for enhanced reaction kinetics for HER and higher stability in alkaline electrolytes as well. Yan et al. prepared FeNi-N nanosheets using electrodeposition technique with carbon fibre cloth as a highly conductive substrate, showing smooth charge transport kinetics and exhibiting overpotential of 106 mV much lesser than Ni-N nanosheets on carbon fibre cloth (155 mV) [236]. It was revealed that the integration of the porous structure and the interconnected nanosheets contributed the enhanced activity and further offered high structural stability of 25 h in 1.0 M KOH solution. Figure 15 illustrates the study of HER activity of different nitride-based materials in comparison with Pt/C and N-CFC using Tafel plots, polarization curve, stability and impedance measurements.

Copper-based MN materials including their hybrids with bimetallic [237], oxynitride [237], carbonate hydroxides [238], and metal phosphides [239] have also proven to be efficient catalysts for HER. In one of the reports, it was demonstrated that the addition of Co-Ni carbonate hydroxides on Cu_3_N, which has a 3D hierarchical structure, significantly lowers the overpotential and the Tafel slope, demonstrating excellent HER activity. The characterization results revealed that the high activity is attributed to multiple reasons including 3D hierarchical structure with abundant active catalytic sites, improved electronic properties and fast reaction kinetics from the highly stable, active and well-developed interface and strong bonding of Cu_3_N and Co-Ni carbonate hydroxides. The combination of 3D architecture and the interface interplay [238]. Using MOF as a template and ammonification at 380°C, Cu_x_Ni_4-x_N were prepared by Gan and co-workers, delivering a low potential of 58 mV and an excellent Tafel slope of 67 mV/dec where a large electrochemical active surface area of 162.9 mF cm^−2^ was observed [237]. It is noteworthy to mention that the prepared bimetallic nitride is rich in defects formed from the Ni and O vacancies while offering higher conductivity and enhanced electronic structure, offering fast kinetics for HER. Not only the defect sites but also the specific surface area and surface wettability also play a critical role in dictating the HER activity of the MN catalysts. For instance, Wang et al. reported Cu_1_Ni_2_-N with a high surface area (61.83 m^2^/g) and electrochemical active surface area of 42.02 mF cm^−2^ and enhanced surface wettability which required an overpotential of only 71.4 mV at 10 mA/cm^2^. The authors found that the enhanced activity is attributed to the combination of high specific surface area, surface wettability and the bifunctional sites introduced by Cu_4_N and Ni_3_N which decreases the d band centre from the Fermi level of the hybrid material [240]. Similarly, niobium-based nitride materials are demonstrated as the HER catalysts [232,241,242]. Zhang et al. reported that Re-Ni nanoparticles coated niobium titanium nitride nanotube arrays delivered a high HER activity with a Tafel slope of 52.9 mV/dec in 1 M KOH solution [232]. Notably, the developed Nb_4_N_5_/TiN materials perform the dual function of being excellent support and interlayer with high conductivity and corrosion resistance. In another report, Li et al. studied the effect of nitridation by ammonia treatment on Nb_2_O_5_ catalyst which delivered faster and stable reaction kinetics of HER activity [241]. Here, the Tafel slope and onset potential improved from 146 mV/dec to 92 mV/dec and 402 to 96.3 mV for Nb_2_O_5_ and Nb_2_N materials where the boosted performance is attributed to the large electrochemically active surface area (8.2 mF cm^−2^) which is 7.7 times better compared to Nb_2_O_5_.

**Figure 16.**
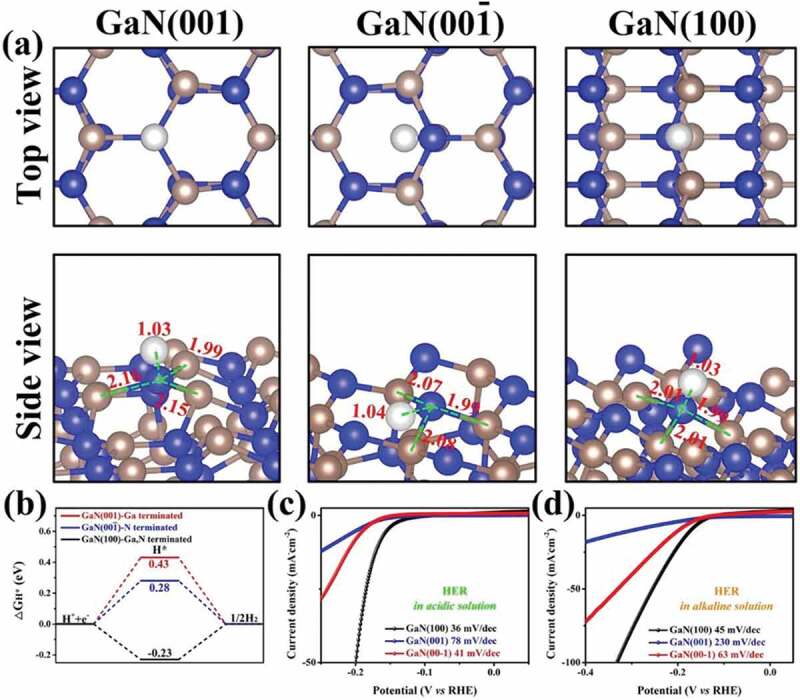
Comparison of three different facets of GaN – GaN(001), GaN(00–1) and GaN(100) (a) Adsorption sites on different materials (b) H* adsorption free energy (c) HER activity in 0.5 M H_2_SO_4_ (d) HER activity in 1.0 MKOH (Reproduced with permission from [244]).

Gallium nitride with different crystal orientations has been engaged in HER application [243,244]. However, the scarcity of Ga and high cost makes gallium-based nitrides make them less attractive for commercialization. For instance, Huang et al. prepared Pt decorated porous GaN using electrodeposition technique as a binder-free catalyst for HER and obtained good catalytic performance, achieving 10 mA cm^−2^ with over potential of 98 mV and stability of 1000 cycles. The Tafel slope of 85 mV dec^−1^ is achieved, which is much lower when compared to that of nonporous Pt decorated GaN (105 mV/dec) [243]. In another interesting study, Hu et al. studied the effect of anisotropy of three different single-crystal GaN facet materials with an overpotential of 168 mV, 234 mV and 205 mV for GaN(100,), GaN(001) and GaN(00–1), respectively, recorded at 10 mA cm^−2^ where the higher catalytic activity of GaN(100) is attributed to the low adsorption-free energy of H* (ΔG_H*_) calculated from DFT measurements [244]. Moreover, the GaN (100) material performed better in acidic media (36 mV/dec, 10 days) with much better stability when compared to alkaline solution (45 mV/dec, 5000 cycles). The comparison of HER activity, adsorption sites and H* adsorption-free energy using the DFT calculations of three different facet GaN materials are illustrated in Figure 16.

From the above examples, it is clear that bimetallic nitrides deliver better HER activity compared to single MNs resulting from the dual active sites from the coordination of two different metals. Further doping with other metals and heteroatoms can improve the conductivity and catalytic active sites for enhanced HER kinetics. Making hybrids or composites with MNs can alter the electronic structure of the parent material as the heterostructures of MN and the functionalizing agent made into a single system improve the HER activity benefitting from the dual active sites [215]. Most importantly, the porous MN materials can outperform the non-porous materials owing to the additional catalytically active sites

## 6. Photocatalytic production of hydrogen

**Figure 17.**
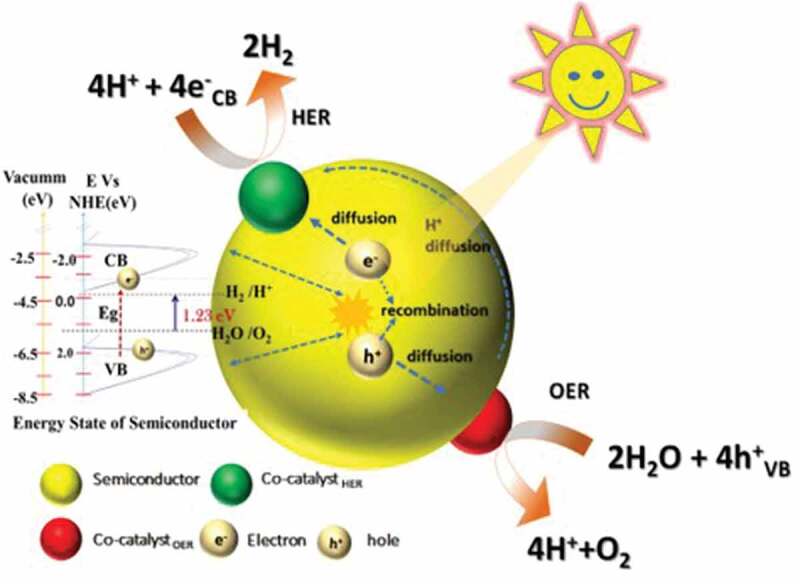
Schematic representation of photocatalytic water splitting reaction, illustrated the fundamental process like charge carrier generation and separation, charge diffusion, recombination, hydrogen evolving reaction, proton (H^+^) transport and oxygen-evolving reaction.

Photocatalytic water splitting reaction is another most attractive option for renewable hydrogen production as the initial feedstock i.e. sunlight and water are inexhaustible, plentiful, and widely distributed. The semiconductor catalyst is the mainspring of photocatalysis that instigates the water-splitting reaction on illumination. The process is thermodynamically uphill, requires a standard free-energy of (ΔG^0^) of 237.2 kJ mol^−1^, or a potential of 1.23 eV [245,246]. Particularly, this much amount of energy can be attained by only those semiconductors having bandgap equivalent or greater than 1.23 eV. In general, semiconductor photocatalytic water splitting involves three main steps: (i) photon absorption to generate electron-hole pair and separation of charge carrier (ii) charge carrier transportation to semiconductor surface or available active sites (iii) utilization of charge carrier to trigger redox reactions [246]. The fundamental process of water splitting with semiconductor photocatalyst is described in Figure 17. The thermodynamic and kinetic of these steps determine the water-splitting efficiency of the photocatalyst. In the initial step of the reaction, the efficiency of electron-hole (carrier) formation is largely determined by the photonic absorptivity of the semiconductor, its chemical composition, and bandwidth [247]. Whereas, separation of charge carriers depends on their lifetime, diffusion length, dimension, and crystalline quality of semiconductors. During the second step of the reaction, photogenerated carriers travelled through the semiconductor-liquid interface to reach reaction active sites present on the surface. The defects present in semiconductor acts as trapping centre wherein the carrier recombination reaction takes place, resulting in loss of energy either in the form of heat or light emission [248]. The particle size of photocatalyst also determined charge carrier separation, for nano-dimension framework the carriers have to travel a smaller distance to reach the surface-active site, resulting in a decreased rate of recombination. Therefore, nanostructured photocatalysts offer high photocatalytic activity compared to bulk [249,250]. Furthermore, near-surface band structure largely influences charge carrier extraction at the semiconductor-liquid interface. Apart from bandgap requirement, the stability of photocatalyst in an aqueous medium is highly essential to realize efficient H_2_ and O_2_ evolution. In addition, co-catalyst is often decorated on semiconductor surface to perform redox reactions with high efficiency. The presence of a co-catalyst on the semiconductor surface ensures the catalytic active sites formation and simultaneously, decreases the activation energy or overpotential of reaction that contributes to charge carrier separation, and inhibits photo-corrosion [251].

Over the decades, numerous potential photocatalysts are extensively developed for overall water splitting reaction but very few of them have realized high activity for H_2_ and O_2_ evolution with promising apparent quantum efficiency under wide visible light illumination. In this context, the MNs family of materials have gained significant attention as a semiconductor photocatalyst for overall water splitting reaction under a broad range of the solar spectrum. The prospects like tunable bandgap, unique electronic structure, high electrical conductivity, and good photo-corrosion resistivity make them different from other conventional oxide-based photocatalysts. Over the years, MNs have been extensively studied for photocatalytic water splitting reactions owing to their advantageous optical, structural, and semiconducting properties. The section appended below will provide a recent overview on MN, mixed MN, and porous MN for hydrogen production under UV and visible light illumination. Table 5 summarizes single, mixed and porous nitride based photocatalysts for H2 and O2 production.

**Table 5.** A brief summary of single, mixed and porous nitride-based photocatalysts for H_2_ and O_2_ production

Photocatalyst	Co-catalyst	Light Source	Reaction solution	H_2_	O_2_	Efficiency	Ref
**MN-assisted photocatalytic hydrogen production**							
*β*-Ge_3_N_4_	RuO_2,_ 1 wt %	450-W high-pressure mercury lamp	1 M H_2_SO_4_ (pH-0)	1 mmol.h^−1^	0.5 mmol.h^−1^	AQE – 9% (300 nm)	[255]
*β*-Ge_3_N_4_	RuO_2_, 1 wt %	450-W high-pressure mercury lamp	1 M H_2_SO_4_	1.10 mmol.h^−1^	0.55 mmol‚h^−1^	-	[256]
GaN (Mg^2+^, Zn^2+^ and Be^2+^) doped)	RuO_2,_ 3.5 wt %	450-W high-pressure mercury lamp	Pure water	0.45 mmol.h^−1^	0.225 mmol.h^−1^	-	[264]
GaN powder	Rh_2y_Cr_y_O_3_	450-W high-pressure mercury lamp	1 M H_2_SO_4_ pH 4.5	18.5 µmol.h-1	9.25 µmol.h-1	0.7 % (300–340 nm)	[263]
*GaN* NW	Rh/Cr_2_O_3_	300 W xenon	Pure water	3.6 µmol.h^−1^g^−1^	1.8 µmol.h^−1^g^−1^	0.5%	[265]
Ta_3_N_5_ nanorod	Rh/Cr_2_O_3_	300 W xenon	Pure water	11 µmol.h-1	5.5 µmol.h-1	AQE- 0.22% (420 nm)	[268]
**Mixed MN-assisted photocatalytic hydrogen production**							
Multiband InGaN/ GaN	Rh/Cr_2_O_3_ core-shell nanoparticles	300 W xenon lamp	Pure water	92 mmol.h^−1^.g^−1^	46 mmol.h^−1^.g^−1^	AQE – 1.86 % (386–405 nm)	[283]
*p*-GaN/*p*-In_0.2_Ga_0.8_N double-band nanowire	Rh/Cr_2_O_3_	300 W xenon lamp	Pure water	3.46 mmol.h^−1^g^−1^	1.69 mmol.h^−1^-g^−1^	AQE-0.3 % (525–600 nm)	[287]
Mg-doped quadruple-band InGaN nanowire	HER- Rh/CrOx OER-CoOx	300 W Xenon lamp with AM 1.5 G filter	Pure water	~1840 µmol.cm^−2^.h^−1^	920 µmol.cm^−2^.h^−1^	STH – 5.2 %	[288]
TaON	Pt	visible light irradiation (420 nm)	Methanol-aqueous solution	20 μmol h ^– 1^	660 μmol h^−1^	AQE – 0.2%	[253]
Distorted – order TaON homojunction	Pt	300 W xenon lamp	TEOA – water solution	25 μmol g^−1^ h^−1^	-	-	[292]
(Ga1*x*Zn*x*)(N1*x*O*x*)	Rh–Cr mixed oxide	300 W xenon lamp	Aqueous H_2_SO_4_ pH 4.5	0.46 mmol.h-1	0.23 mmol.h-1	AQE – 2.5% (420–440 nm)	[293]
(Ga_0.82_Zn_0.18_)(N_0.82_O_0.18_)	Rh–Cr mixed oxide	300 W xenon lamp	Aqueous H_2_SO_4_ pH 4.5	3090 µmol.h^−1^g^−1^	1533 µmol.h^−1^g^−1^	AQE-5.9 % (420–440 nm)	[294]
(Ga_0.82_Zn_0.18_)(N_0.82_O_0.18_)	Rh–Cr mixed oxide	300 W xenon lamp	Aqueous H_2_SO_4_ pH 4.5	1093 µmol.h^−1^g^−1^	546.5 µmol.h^−1^g^−1^	AQE-17.3% (400 nm)	[295]
**Porous MN-assisted photocatalytic hydrogen production**							
High Surface area active- Ta_3_N_5_	Pt	High-pressure Hg lamp 450 W	MeOH – water	136 µmol.h^−1^g^−1^	-	-	[299]
Macroporous Ta_2_O_5_	-	300 W Xe lamp	Methanol-water	8.5 µmol h^−1^	-	-	[300]
GaZnON–rGO	Rh	100 W high pressure UV-vis Hg lamp	Methanol aqueous solution	60 µmol h-1	NA	AQE- 2.5 %	[304]

**Note**- H_2-_ Hydrogen gas; O_2-_Oxygen gas; AQE- Apparent quantum efficiency; STH-solar to hydrogen conversion efficiency; *β*-Ge_3_N_4 –_ *β*-Germanium nitride; RuO_2 –_ Ruthenium oxide; GaN-Gallium nitride; Rh_2y_Cr_y_O_3-_ Rhodium–chromium mixed-oxides; Rh/Cr_2_O_3_ – rhodium core and chromium oxide shell structure; Ta_3_N_5-_ tantalum nitride; Multiband InGaN/GaN- Multiband Indium Gallium Nitride/Gallium Nitride; *p*-GaN/*p*-In_0.2_Ga_0.8_N – *p*-type gallium nitride/indium gallium nitride; Mg-doped quadruple-band InGaN nanowire – Magnesium-doped Indium-Gallium Nitride quadruple band; HER – Rh/CrOx – Hydrogen Evolution Reaction Co-catalyst – Rhodium Core and Chromium Oxide Shell; OER-CoOx – Oxygen Evolution Reaction Co-catalyst – Cobalt Oxide nanoparticles; TaON – tantalum oxynitride; Pt – Platinum nanoparticles; TaON homojunction – Tantalum oxynitride homojunction; TEOA – Triethanolamine; (Ga1*x*Zn*x*)(N1*x*O*x*) – solid solution of gallium and zinc nitrogen oxide; Rh–Cr mixed oxide – Rhodium-Chromium mixed oxide nanoparticles; H_2_SO_4 –_ Sulphuric acid; Ta_3_N_5 –_ tantalum nitride; Ta_2_O_5 –_ tantalum oxide; GaZnON–rGO – Gallium–zinc oxynitride-reduced graphene oxide; Rh – Rhodium.

### 6.1 MN-assisted photochemical hydrogen production

**Figure 18.**
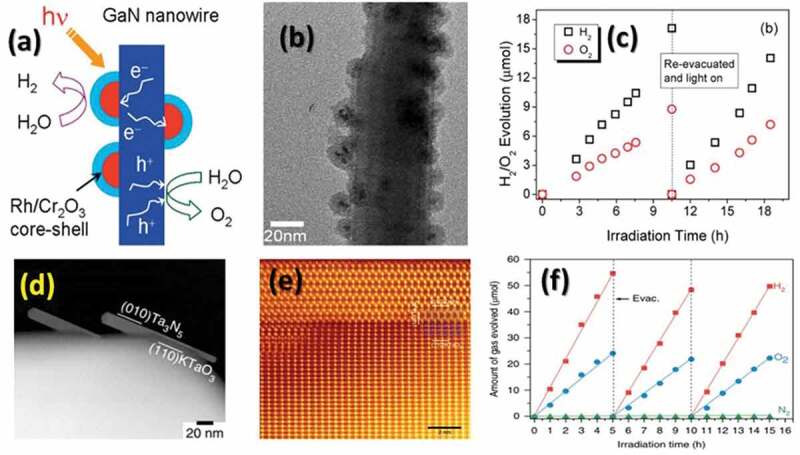
**(a)** Schematic representation of water splitting on Rh/Cr_2_O_3_ core-shell nanostructures decorated GaN nanowires **(b)** Low magnification TEM image of Rh/Cr_2_O_3_ /GaN composites illustrated uniform distribution of the Rh/Cr_2_O_3_ nanoparticles on GaN nanowire surfaces. **(c)** Plot of H_2_ and O_2_ evolution as function of time for Rh/Cr_2_O_3_ /GaN composite under a 300 W xenon lamp irradiation (Reproduced with permission from [265]) **(d**) ADF-STEM images of Ta_3_N_5_/KTaO_3_ composite illustrated the growth direction of the Ta_3_N **(e)** Colorized and magnified ADF-STEM images of a Ta_3_N_5_ nanorod in Ta_3_N_5_/KTaO3 **(f)** Plot of stoichiometric H_2_ and O_2_ evolution for Rh/Cr_2_O_3_-modified Ta_3_N_5_/KTaO_3_ assisted water splitting under visible light (λ ≥ 420 nm) (Reproduced with permission from [268]).

Development of MN-based photocatalyst started with oxynitrides like Ta_2_N_3_ [252], TaON [253], and LaTiO_2_N [254], and then shifted to MN after the first successful demonstration of single-phase *β*-Ge_3_N_4_ [255] for photocatalytic hydrogen and oxygen evolution. The RuO_2_ nanoparticles (20–50 nm) decorated *β*-Ge_3_N_4_ have shown stoichiometric H_2_ and O_2_ evolution under UV light illumination with an apparent quantum efficiency (AQE) of 9%. The plane-wave DFT calculations revealed that valance band holes (N2p) contributed toward the photooxidation of water (O_2_) without assistance from any OER co-catalyst while RuO_2_ nanoparticles served as HER co-catalyst. The photocatalytic performance of RuO_2_/*β*-Ge_3_N_4_ depends on pH, and the highest activity was recorded with 1 M H_2_SO_4_ solution. The optimal acid concentration suppresses the hydrolysis of metallic components and simultaneously promotes H_2_ production [256]. Subsequently, a fourfold improvement in photocatalytic performance of *β*-Ge_3_N_4_ was observed through high-temperature ammonia treatment of bulk powder. The increased photocatalytic activity is attributed to the reduction of anion defects present in bulk and surface [257]. These pivotal results have triggered the investigation of gallium nitride (GaN)-based composite for photocatalytic hydrogen production. Subsequently, GaN-based composites were widely investigated for overall water splitting performance. In 1995, the Turner group [258] first recognized *GaN* potential for water splitting reactions, followed by a number of reports demonstrating its thermodynamic and kinetics prospects for overall water splitting reactions [259–264]. Although, GaN exhibits a limited photoactivity owing to a wide bandgap (3.5 eV) but showed competent photocatalytic H_2_ and O_2_ evolution when decorated with HER co-catalysts like RuO_2_ [260,264] or Rh/Cr_2_O_3_ [263]. Like *β-Ge_3_*N_4_, no OER co-catalyst was required of oxygen evolution which indicated nitrides inherent activity toward photooxidation. Further studies have demonstrated that doping of divalent ions (Zn^2+^, Mg^2+^) has significantly improved overall water splitting potential and chemical stability. Especially, the doping of Mg^2+^ ions elevates the rate of photocatalytic hydrogen production due to formation of Mg_Ga_ acceptor level higher by 0.25 eV above valance band edge that increases the hole concentrations, resulting in high electron mobility to promote reduction reaction [262,264]. On the other side, to avail prospects of nano-dimensions, Mi et al. [265] first time developed GaN nanowires by plasma-assisted MBE technique and recorded higher photocatalytic water splitting activity compared to powder and planer samples. The Rh/Cr_2_O_3_ co-catalyst decorated *GaN* NW has shown the stoichiometric H_2_ and O_2_ evolution under 300 W xenon lamp illumination, schematic representation of GaN/ Rh/Cr_2_O_3_ shown in Figure 18(a). The uniform distribution of Rh/Cr_2_O_3_ on GaN surface was further confirmed with transmission electron microscopy (TEM) investigation (Figure 18(b)). Compared to powder GaN, enhancement in hydrogen production was attributed to large surface-to-volume ratio, increased charge transport, and significantly improved light absorption (Figure 18(c)). They concluded that *Ga*-terminated surface sites actively determined the cleavage of water molecules that are abundant in the one-dimensional nanowire structure compared to powder or planer form [266].

Apart from group-III MN, tantalum nitride (T_3_N_5_) is the commonly studied transition MN owing to promising visible light active bandgap (2.1 eV) and favorable band edge positions to straddle water redox potential [252,267]. Domen group first identified its thermodynamic and kinetic potential for hydrogen and oxygen generation in the presence of suitable co-catalyst and sacrificial donor under visible light illumination [252]. Despite the advantageous electronic structure, very low quantum efficiency has been reported with Ta_3_N_5_ due to the high density of recombination centres developed during the nitridation process. Wang et al. [268] demonstrated the overall water splitting with single-crystal Ta_3_N_5_ nanorod decorated with Rh/Cr_2_O_3_ co-catalyst. They found that the growth of defect-free Ta_3_N_5_ nanorod on cubic KTaO_3_ particles significantly promotes the overall water splitting reaction. Figures 18d and **18e** represent the spatial arrangement of Ta_3_N_5_ nanorod on KTaO_3_ crystal, indicating a regular arrangement of atoms from surface to interior without any defects or grain boundaries. The trend of H_2_ and O_2_ production with Ta_3_N_5_ nanorod is shown in Figure 18f. A similar group studied the correlation between synthesis condition, structural defects, carrier transport dynamics, and water splitting activity with Ta_3_N_5_ using ultrafast transient absorption spectroscopy [269]. This work demonstrated the importance of defect-free single crystal for photocatalytic water splitting reaction.

Besides, transition MNs (TMNs) are widely explored as co-catalyst for hydrogen evolving reactions owing to metal-like characteristics. It is a class of interstitial nitride wherein intercalated nitrogen atom expands the parent lattice structure, which reduces the inter-atomic interactions. Simultaneously, contracted metallic d-band increases the density of state near Fermi energy level. These changes alter the existing electronic structure and improve the density of valance electrons even better than the parent metal. This feature has tuned the M-H bond strength to an optimal range where hydrogen desorption takes place from the nitride surface and that makes the nitrides a potential co-catalyst [171,270]. Recently, TMN such as Co_3_N, Fe_2_N, Ni_3_N, Mo_2_N, and NbN are extensively explored as co-catalyst for photocatalytic hydrogen evolution reaction [171]. For instance, Xu et al. [271] have developed Co_3_N decorated Cd_0.5_Zn_0.5_S composite and achieved the highest sacrificial-assisted hydrogen production of 160.7 mmol h^−1^g^−1^, 125 times higher than pure composite under visible light illumination (420 nm). The high activity was obtained through the low-lying conduction band of Co_3_N and its excellent electronegativity provide efficient electron capture ability that ensures the separation of photoexcited holes. In addition, a tight co-catalyst and semiconductor interface promotes electron transport, and hence, Co_3_N accelerates the hydrogen evolving reaction. In photocatalysis, nickel nitride (Ni_3_N) presents an excellent activity as hydrogen evolving co-catalyst. DFT calculations suggested that the Ni_3_N have electronic energy states crossing Fermi level which is as similar as Ni metal. It also substantiated its metallic character and proved its outstanding potential for electrons transport during water-splitting reaction. Simultaneously, hydrogen adsorption free energy (ΔG_H*_) of −0.17 eV (closes to zero) describes its hydrogen evolution activity compared to Ni metal (i.e. ΔG_H*_ = −0.26 eV), although it is far from Pt (ΔG_H*_ = −0.09 eV) [272]. The potent of Ni_3_N as a co-catalyst has been experimentally illustrated with Ni_3_N/2D-C_3_N_4_ nanocomposite, the obtained HER of ∼1347.8 μmol/g/h is higher than Ni-based or even many noble metals-based C_3_N_4_ composite [273]. In addition, Yang et al. [274] have demonstrated photocatalytic water splitting with Ni_3_N-Au-TiO_2_ composite with ultralow Au concentration of 0.00025 wt% which exhibits HER of 86.7 μmolg^−1^ h^−1^ even higher than Pt-TiO_2_ (73.4 μmolg^−1^ h^−1^). The enhancement is attributed to the synergistic effect of Ni_3_N and Au nanocluster that accelerated the charge separation and transport phenomenon. It indicates that Ni_3_N as an efficient co-catalyst can decrease the noble metal loading significantly for photocatalytic hydrogen evolving reaction. Furthermore, MNs like Fe_2_N [275], Mo_3_N [276] and VN [277] were also demonstrated as efficient co-catalysts for hydrogen evolution by facilitating better interfacial charge transport, catalyzing H_2_ production at low overpotential, and accelerating hydrogen production kinetics. These examples promote the cost-effective rational designing of novel MN as co-catalyst to reduce noble metal utilization for photocatalytic hydrogen production.

### 6.2 Mixed MN-assisted photochemical hydrogen production

**Figure 19.**
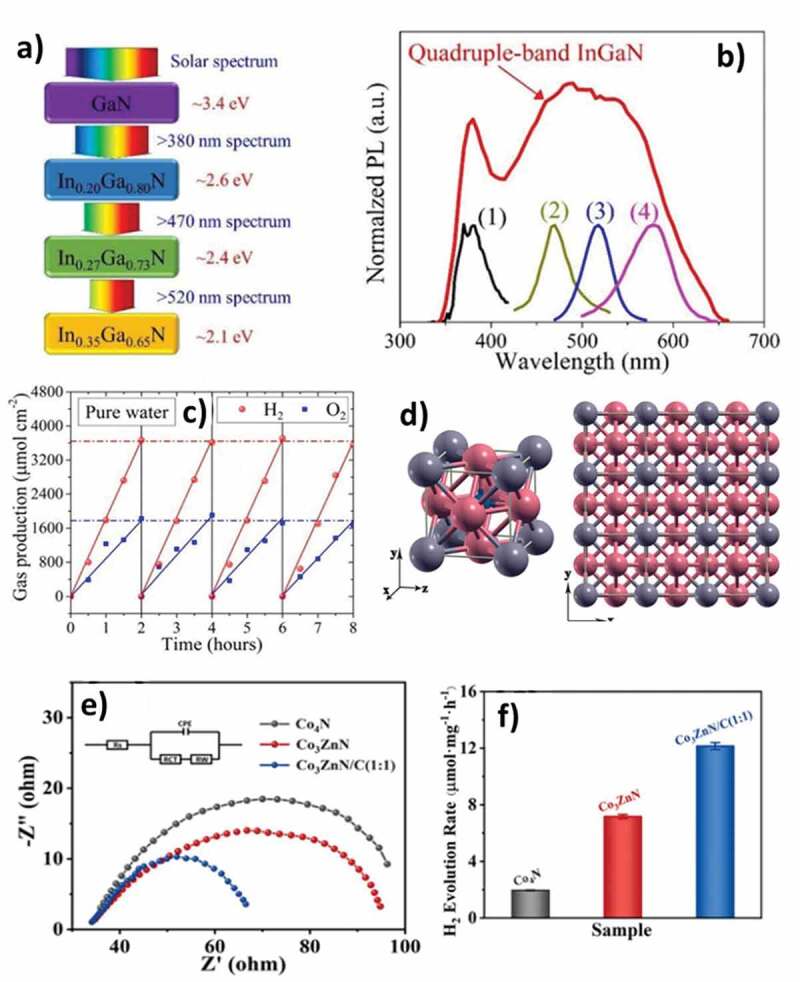
**(a)** Schematic of quadruple-band InGaN nanowire, illustrating the light absorption process on the multi-band **InGaN** stacks **(b)** Room-temperature PL spectrum of the quadruple-band InGaN nanowires, includes PL of (1) p-GaN, (2) p-In_0.20_Ga_0.80_N, (3) p-In_0.27_Ga_0.73_N, and (4) p-In_0.35_Ga_0.65_N. **(c)** Plot of H_2_ and O_2_ gas generation as a function with quadruple InGaN with HER and OER co-catalyst, (Reproduced with permission from [288]) **(d)** The atomic structure model of Co_3_ZnN (e) Electrochemical impedance spectra of Co_4_N, Co_3_ZnN and Co_3_ZnN/C (f) The average rates of H_2_ evolution under visible-light (λ > 400 nm) over as-prepared Co_4_N, Co_3_ZnN and Co_3_ZnN/C (1:1) samples in Eosin Y-TEOA system, (Reproduced with permission from [289]).

Mixed MN is another most important class of nitride wherein the energy bandgap of material can encompass the entire solar spectrum by tuning the aspect ratio between two metals. In this category, indium-gallium nitride (*InGaN*) heterostructures with higher *In* content (up to 40–50%) have gained significant attention owing to tunable energy gap from ultraviolet to infrared region and favorable conduction and valance band edge positions to straddle water redox potential [278,279]. However, the development of high crystalline InGaN with higher In content is quite challenging due to the difference in formation enthalpies and lattice mismatch between InN and GaN [279,280]. These factors cause surface segregation, resulting in the formation of numerous non-radiative recombination centres. Among reported different growth methodologies, plasma-assisted MBE technique ensures the formation of defect free indium (In) rich InGaN structure with high crystallinity to function as visible light active catalyst [281,282]. Kibria et al. [283] have developed multiband InGaN/GaN nanowire heterostructure grown on vertically aligned GaN template, consist of ten self-organized InGaN/GaN quantum dots (In composition of ∼15-50%) well separated from InGaN nanowire segment (In composition of ∼ 11%) and upper GaN segment was p-doped with Mg. This triple band heterostructure corresponds to band gap of 2.22, 2.96 and 3.4 eV, which triggers the water splitting reaction in green, blue, and UV light spectrum. The triple band InGaN/GaN nanowire decorated with Rh/Cr_2_O_3_ core-shell nanoparticles has demonstrated stable hydrogen production of ~92 mmolh^−1^g^−1^ with the highest AQE of ~ 1.86% (Figure 19c). It observed that controlled growth of InGaN quantum dots and ternary wire segments synergistically contributed toward surface properties, reduced photoexcited charge carrier recombination and provided excellent chemical stability. As an alternative, the dye-sensitized InGaN/Rh heterostructure also showed photocatalytic hydrogen production under green, yellow, and orange light irradiation with AQE of 0.3% and represents a viable approach to harness deep visible light illumination without increasing in content [284]. It is clearly demonstrated that controlled Mg-doping (*p*-type) can effectively control charge carrier transport near semiconductor-electrolyte interface by optimizing surface band bending [285,286]. The *p*-GaN/*p*-In_0.2_Ga_0.8_N double-band nanowire heterostructure achieved nearly 30 times higher hydrogen production compared to undoped material, wherein near-surface bending of GaN and InGaN counterparts was optimized using Mg doping [287]. This double band nanowire decorated with Rh/Cr_2_O_3_ co-catalyst has shown apparent quantum efficiency of 12.3% in 400–475 nm range and exhibits solar to hydrogen efficiency of 1.8% under concentrated sunlight. Recently, Mg-doped quadruple-band InGaN nanowire heterostructure [288] was developed on Si wafer by plasma-assisted MBE and tested its photocatalytic water splitting performance under xenon lamp irradiation with AM 1.5 G filter. The schematic of the as-grown quadruple-band InGaN nanowire is represented in Figure 19a. The broad PL emission spectrum indicates its solar absorptivity across the entire visible light spectrum (Figure 19b). The trend of H_2_ and O_2_ evolution is represented in Figure 19c. This quadruple-band nanowire heterostructure has shown the highest solar-to-hydrogen (STH) conversion efficiency of 5.2% through (i) enhanced visible light absorption with quadruple-band structure, (ii) nonplanar wafer-assisted light absorption and trapping effect, and (iii) controlled Mg^+^ ion doping promotes electron-hole separation and extraction.

Apart from conventional group-III MNs, some transition metal-based mixed (binary and ternary) MNs have been reported for potential hydrogen production. For instance, anti-perovskite (Figure 19d) type cobalt zinc nitride (Co_3_ZnN) [289] synthesized by replacement of cobalt by zinc atom have shown optimistic sacrificial-assisted HER of 15.4 μmol.mg^− 1^h^− 1^(Co_3_ZnN/C), six times higher than monometallic Co_4_N phase (Figure 19f). This enhancement is attributed to more negative d-band centre (−1.87 eV) and low-lying antibonding energy state that facilitates facile desorption of hydrogen from catalytic surface. In addition, Co_3_ZnN/C showed improved charge transport compared to Co_3_ZnN and Co_4_N, illustrated with electrochemical impedance spectra (Figure 19e). The prospect of multicomponent MN for hydrogen evolution was further demonstrated with Chromium-Titanium nitride (Cr_0.5_Ti_0.5_N)/CdS nanocomposite wherein Cr_0.5_Ti_0.5_N has boosted the rate of hydrogen production (2.44 mmolg^−1^h^−1^), which is 120 times higher than pure CdS or even better than Pt-CdS composite (2.04 mmolg^−1^h^−1^) [34]. These experimental results have demonstrated the utility of transition mixed MN for water splitting reaction with possible replacement of noble metal catalyst.

Metal oxynitride is an important class of mixed MN that combines the advantages of oxides and nitride. The oxynitrides are well studied for catalytic applications owing good electrical conductivities and corrosion resistivity. The valance band of these materials is populated with hybridized N_2p_ and O_2p_ orbitals with higher N_2p_ contribution, whereas the conduction band composed of empty d-orbital of the respective metal ion. These hybridized orbitals have higher energy O_2p_ orbital and hence, the oxynitride has smaller band gap than the respective oxide. This feature makes the oxynitride as a potential candidate for photocatalytic water splitting reaction [290,291]. Domen et al. have demonstrated the efficient photocatalytic water oxidation (oxygen evolution) on TaON surface with Ag^+^ as a sacrificial agent under visible light illumination with an estimated quantum yield of 34 % [253]. However, they reported a very low photocatalytic hydrogen production (quantum efficiency 0.2%). On the contrary, disordered *TaON* with surface oxygen vacancies is more active for photocatalytic hydrogen production (25 µmol^−1^h^−1^g^−1^) than pure structure (11 µmol^−1^h^−1^g^−1^) [292]. The crystallinity of TaON particles was reduced with increment in oxygen vacancies, which led to the formation of a crystalline core and an amorphous shell structure. This disorder–order homojunction limits the photogenerated charge carrier recombination and promotes surface photocatalytic redox reactions.

Further, in this type, Gallium-zinc oxynitride i.e. a solid solution of GaN and ZnO is observed as most efficient in overall water splitting and efficient hydrogen production under visible light spectrum. The band gap of (Ga*_1-x_*Zn*_x_*)(N_1-x_O_x_) is estimated as 2.6–2.8 eV which is substantially lower than individual GaN (3.4 eV) and ZnO (3.2 eV). Theoretical investigation revealed that smaller band gap is due to the repulsion between p (N2p) and d (Zn3d) orbital electrons of valance band. Maeda group have provided a detailed investigation on GaN:ZnO solid solution-mediated photocatalytic water splitting under visible irradiation (∼510 nm) [293]. For overall water splitting, they reported the highest AQE of 2.5 % for Rh_2-y_Cr_y_O_3_ decorated on (Ga_1-x_Zn_x_) (N_1-x_O_x_) composite with 420–440 nm. The Rh-Cr-O mix oxide nanoparticle trap the excited electron and hole, in which Rh^+3^ provides hydrogen evolution active site and Cr^3+^ oxides shell prevents back reaction. In follow-up study, the apparent quantum efficiency of 5.9% (420–440 nm) is achieved through post calcination of as-synthesized (Ga_1-x_Zn_x_) (N_1-x_O_x_) composite. The post-treatment is observed to inhibit the charge carrier recombination by reducing the zinc- and/or oxygen-related defects [294]. Li et al. was further improved AQE to 17.3% at 400 nm through (Ga_1-x_Zn_x_) (N_1-x_O_x_) nanostructure with grain size of 6 nm [295]. Recently, there is a significant progress seen in tantalum and niobium-based perovskite oxynitride material which interestingly offers the bandgap in 1.5–2.5 eV range that will help achieve the water-splitting reaction in the wide visible light region [296].

### 5.3 Porous MN

**Figure 20.**
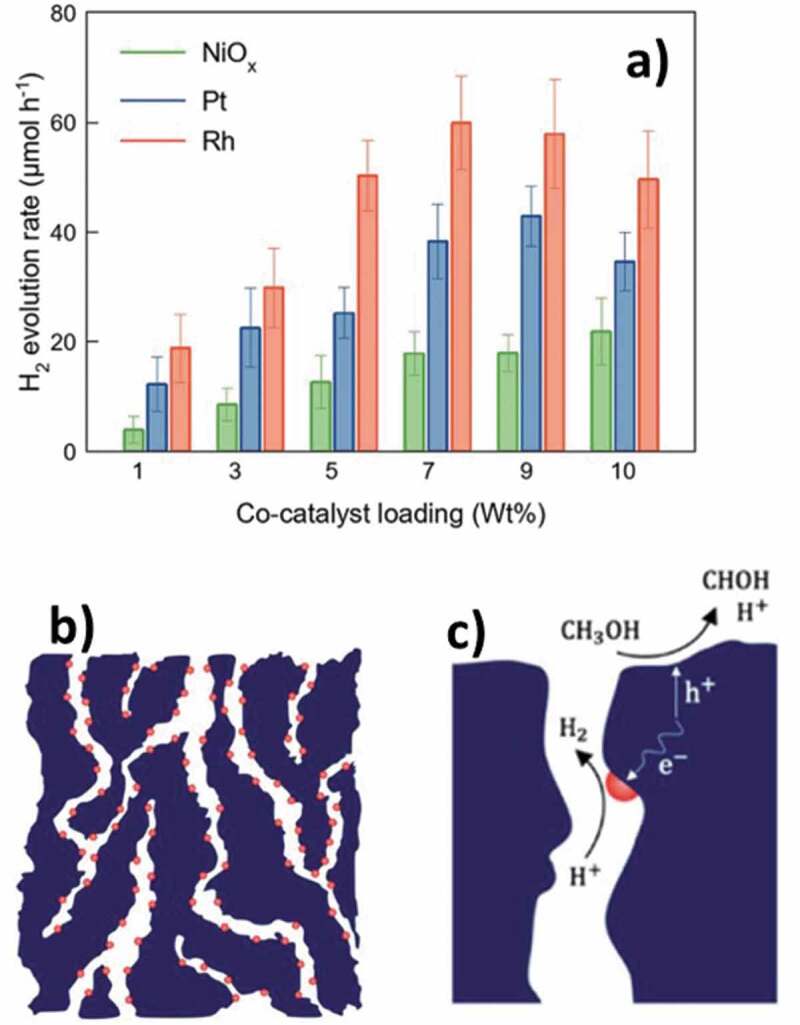
**(a)** Plot of hydrogen evolution of the nanoporous GaZnON photocatalyst decorated with various amounts of NiOx, Pt, and Rh co-catalysts in 10 V% methanol aqueous solution. (b) Schematically represented the deposition of co-catalyst in structural nanopores and (c) Electron and hole diffusion toward the active sites positioned in structural nanopores; (Reproduced with permission from [304]).

Porous MN is the new emerging class of materials in which the synergy of porous microstructure and semiconducting nitride<apos;>s characteristic opens new opportunities for the design and development of efficient photocatalyst. In general, the porous structure offers a high specific surface area with abundant channels, easy accessibility to guest molecules, permits facile electron/ion transport and mass diffusion, assists catalytic active site formation, lowers material corrosion rate, and provides more stability [297]. In addition, promoted photon harvesting is observed with porous materials owing to enhanced light scattering within elongated pores [298]. In general, MNs with the aforementioned structural merit exhibited higher rate of photocatalytic hydrogen production compared to non-porous material. For instance, tantalum nitride (Ta_3_N_5_) with the high surface area of 61 m^2^/g derived using mesoporous carbon nitride as a template has shown HER of ∼ 27.2 µmol.h^−1^ on visible light illumination, which was higher than reference material (∼2.92 µmol.h^−1^) [299]. A similar observation was also recorded with macroporous tantalum oxynitride (TaON) and tantalum nitride (Ta_3_N_5_), wherein macroporosity offers an additional benefit in photonic absorption [300]. They marked that photocatalytic reaction in porous material has occurred throughout the volume which is not restricted to the outer surface only. For the synthesis of porous semiconductor material, the metal-organic framework is considered as an ideal precursor. Using this approach, the porous iron nitride nanocubes (Fe_2_N) has been derived from Prussian blue-MOF and tested for water splitting reaction under illumination of 300 W xenon lamp with Eosin-Y used as the photosensitizer. It showed sacrificial-assisted hydrogen production of 14.5 mmol.h^−1^.g^−1^ [301]. This optimistic water splitting activity is due to the free availability of *Fe*-3d electrons around Fermi energy level and its porous microstructure endowed material with highly dense HER active sites. Furthermore, the porosity along with the three-dimensional MN framework exhibits higher water-splitting ability owing to morphological merits like high specific surface area, the short diffusion length of photogenerated charge carriers to HER active site, and facile mass transport in a hierarchical pores [302,303]. To avail these structural merits, Taghipour *et al*. [304] have engineered a nanoporous 3D-GaZnON–rGO composite and found a nearly 7.5-fold increment in hydrogen production compared to conventional 1 wt% Rh-loaded photocatalyst (GaN:ZnO) on visible light irradiation. The water-splitting activity is improved through shorting of charge carrier diffusion length and enhancement in HER active sites within the quasi-three-dimensional nanostructure. The rate of hydrogen production with respect to different co-catalyst loading is shown in Figure 20a. The synergy of 3D microstructure with uniform nanoporous morphology increases the GaN:ZnO specific surface area up to 61 m^2^/g, which is much higher than bulk counterpart. In addition, the nanoporous structure offers more additional sites for co-catalyst deposition, as schematically represented in Figure 20b, and further reduces shading of photocatalyst surface and offers more area for photonic absorption. Simultaneously, the presence of co-catalyst on nanopores significantly reduces the rate of charge carrier recombination that occurred at bulk, due to shorting of carrier diffusion length (Figure 20c). These morphological benefits of porous nitride ensure a high rate of photocatalytic hydrogen production than bulk powder analog. Additionally, they also provide a general framework for engineering porous photocatalyst with numerous HER active site with enhanced charge and mass transport process.

## 7. Conclusions

The review covered the general introduction to MNs in both non-porous and porous forms, different methods of synthesis, and their application perspective for hydrogen generation via electrochemical and photocatalytic pathways. MNs have been extensively explored as catalysts for these applications and their application scope is expected to widen with the discovery of new materials with novel properties. Out of several synthesis strategies, thermal annealing under an ammonia atmosphere is one of the well-established techniques. The variation in the properties and structural modifications could be made possible through a combination of thermal annealing with other procedures such as hydrothermal treatment, solvent evaporation, molten salt route etc. Magnetic sputtering, electrospinning, and electrodeposition methods could also be used for synthesizing specific forms of the MNs. In general, the MNs display low porosity which is counted in terms of diminished surface area and negligible pore volume. However, the non-porous MNs show good activity for the intended applications of electrochemical and photocatalytic production of hydrogen. It needs to be mentioned that introducing porosity in MNs has been explored on few occasions; however, the surface area and pore volume values are low to moderate in comparison with materials such as MOFs, zeolites, or porous carbons. The review elaborates the specific synthesis methods that can be employed for the fabrication of MNs in their porous forms which include the use of a reactive template such as carbon nitrides, and the use of porous supports such as carbon, graphene, and carbon nanotubes. Such porous nitrides and their synthesis methods certainly deserve more attention from the research community owing to their unique physico-chemical properties and enhanced performance for catalytic applications.

The field of MNs needs to be better understood to reveal their full potential for various applications. For example, in situ characterization tools could be employed to study the aspects related to their synthesis and application performance. More studies on synthesizing MNs in both non-porous and porous forms with variable particle size, different morphologies, high porosity, facile dispersion of nanoparticles should be performed to evaluate their application effectiveness. The porosity is a crucial aspect and more developments in the reactive/sacrificial templating, thermal annealing with porous support, and molten salt route for the synthesis of porous MNs need to be made. From an application point of view, MNs are perfectly suited for hydrogen generation via electrochemical and photocatalytic pathways. The porous MNs with unique morphology, surface functionalities and tunable band gap are worth exploring for hydrogen generation.

Overall the key merits of MNs for hydrogen production include their attractive physico-chemical properties such as tunable band gap, high surface area, controllable size and shape, and surface functionalization. The review discussed the fundamentals of the synthesis of MNs and at the same time provides a thorough understanding of their role toward their efficacy for these applications. The future of MN is bright and further research to gain more understanding of various aspects of their synthesis, properties, structure, and application evaluation will make a strong case for their production at an industrial scale level.
